# Mechanism of GAPDH Redox Signaling by H_2_O_2_ Activation of a Two−Cysteine Switch

**DOI:** 10.3390/ijms23094604

**Published:** 2022-04-21

**Authors:** Paul A. Hyslop, Michael O. Chaney

**Affiliations:** 1 Arkley Research Labs, Arkley BioTek, LLC, 4444 Decatur Blvd., Indianapolis, IN 46241, USA; 2 Eli Lilly Research Laboratories, Lilly Corporate Center, Indianapolis, IN 46285, USA; chaney1643@comcast.net

**Keywords:** oxidative stress, redox signaling, glyceraldehyde−3−phosphate dehydrogenase, hydrogen peroxide, two−cysteine redox switch, thiosulfinic ester, thiosulfonic ester, neurodegenerative disease, Molecular Dynamic Simulation

## Abstract

Oxidation of glyceraldehyde−3−phosphate dehydrogenase (GAPDH) by reactive oxygen species such as H_2_O_2_ activate pleiotropic signaling pathways is associated with pathophysiological cell fate decisions. Oxidized GAPDH binds chaperone proteins with translocation of the complex to the nucleus and mitochondria initiating autophagy and cellular apoptosis. In this study, we establish the mechanism by which H_2_O_2_−oxidized GAPDH subunits undergo a subunit conformational rearrangement. H_2_O_2_ oxidizes both the catalytic cysteine and a vicinal cysteine (four residues downstream) to their respective sulfenic acids. A ‘two−cysteine switch’ is activated, whereby the sulfenic acids irreversibly condense to an intrachain thiosulfinic ester resulting in a major metastable subunit conformational rearrangement. All four subunits of the homotetramer are uniformly and independently oxidized by H_2_O_2_, and the oxidized homotetramer is stabilized at low temperatures. Over time, subunits unfold forming disulfide−linked aggregates with the catalytic cysteine oxidized to a sulfinic acid, resulting from thiosulfinic ester hydrolysis via the highly reactive thiosulfonic ester intermediate. Molecular Dynamic Simulations provide additional mechanistic insights linking GAPDH subunit oxidation with generating a putative signaling conformer. The low−temperature stability of the H_2_O_2_−oxidized subunit conformer provides an operable framework to study mechanisms associated with gain−of−function activities of oxidized GAPDH to identify novel targets for the treatment of neurodegenerative diseases.

## 1. Introduction

H_2_O_2_ is an essential redox cofactor constitutively produced by cell metabolism, primarily by the NAD(P)H oxidases of immune cells and mitochondria. H_2_O_2_ produced by innate immune cells is a potent bacteriostatic agent contributing to host defense [1,2]. In vitro and in vivo pathological levels of various reactive oxygen species (ROS) contribute to apoptotic and necrotic cell death [3,4] via specific signaling mechanisms [5,6]. Following redox modification GAPDH forms complexes with chaperone proteins that are translocated to both nucleus and mitochondria [7,8,9,10,11,12,13], binds and activates enzymes [14], and a substrate for post−translational enzymatic modification [15]. Very little is known regarding how these modifications induce and modulate the structure of the signaling conformer and initiate proapoptotic functions of oxidized GAPDH directly involved in the pathophysiology of neurodegenerative diseases [16,17,18,19,20,21,22]. Recently, a high correlation of S−glutathionylated GAPDH in the blood correlated with the progression of Alzheimer’s Disease (AD) [23], and GAPDH was identified as one of four hub genes associated with AD in a gene profiling study [24].

Different species of ROS modify aspects of GAPDH−induced redox signaling by a variety of mechanisms. Nitrosative stress results in S−nitrosylation of the active site catalytic cysteine residue (C*_c_*SH), which is sufficient to induce GAPDH subunit binding to the chaperone protein Siah−1, and translocation of the complex to the nucleus, initiating apoptosis [25,26,27,28]. H_2_O_2_ initially oxidizes C*_c_*SH residue initially to a stabilized sulfenic acid (C*_c_*SOH), which is readily reduced by excess thiol and reactivates enzyme activity [29,30]. C*_c_*(SOH) can be further oxidized by H_2_O_2_ to cysteine sulfinic (C*_c_*SO_2_H) and sulfonic acids (C*_c_*SO_3_H), both refractory to thiol reduction and enzyme reactivation [31,32,33,34] and is thought to represent one mechanism for redox signaling by GAPDH. However, the subunit crystal structures of GAPDH of native and subunits modified to cysteine sulfonic acid are isomorphous [35], presenting a conundrum for defining a mechanism for how oxidation of the catalytic cysteine residue of GAPDH promotes association with the variety of identified chaperone proteins [36].

Another observation linking H_2_O_2_ oxidation of GAPDH potentially resulting in a signaling conformer is that C*_c_*(SOH) can form an intrasubunit disulfide bond via nucleophilic attack by an almost universally conserved vicinal cysteine C*_v_*(SH), four residues downstream of C*_c_*(SH). It is difficult to explain how disulfide bond formation could compete with the reduction of C*_c_*(SOH) and reactivation of dehydrogenase activity by cellular glutathione (GSH), given the established stability of C*_c_*(SOH) in purified GAPDH in vitro in the absence of thiol(>0.5 h) [30]. This observation has strong mechanistic support from *in silico* Molecular Dynamic Simulations (MDS) demonstrating that the 9Å spatial separation between C*_c_*(SOH) and C*_v_*(SH) is stabilized, and in addition, local steric effects strongly hinder the approach between the cysteine sulfur atoms [34]. It should be noted the active site intrachain disulfide was detected following S−glutathionylation of GAPDH [37], although this result appears to be somewhat controversial, at least in vitro [38]. C*_v_*(SH) has been suggested to play no direct role in the GAPDH oxidation mechanism whereby the enzyme is irreversibly inactivated by H_2_O_2_ [34], although the presence of C*_v_*(SH) is essential for GAPDH activation of endonuclease APE1 regulating DNA repair and transcriptional factors during oxidative stress [39].

In this study, we examine in detail various aspects of the H_2_O_2_ oxidation process of GAPDH using a variety of experimental conditions, probing the kinetics of chemical and biophysical transformations within oxidized subunits, in order to consolidate the disparate observations associated with GAPDH and H_2_O_2_ redox signaling. We demonstrate for the first time that sequential oxidation of C*_c_*(SH) and C*_v_*(SH) by H_2_O_2_ is critical for redox−active participants mediating the irreversible inactivation of GAPDH and formation of a putative metastable subunit signaling conformer. We identify additional redox pathways that link these events with the concomitant formation of the active site sulfonated cysteine, intra and intersubunit disulfide bonding, subunit unfolding, and aggregation [12].

## 2. Results

### 2.1. Stoichiometry and pH−Dependent Kinetics of GAPDH Oxidation

In order to establish the identity of oxidizable residues and their relative subunit distribution within the GAPDH homotetramer, it is first necessary to measure the stoichiometric ratio of H_2_O_2_ consumed oxidizing one mol GAPDH tetramer and the kinetic constants associated with the overall oxidation process. The most facile H_2_O_2_ oxidation process within proteins involves the heterolytic cleavage of the dioxygen bond by nucleophilic attack by the cysteine anion, where the reaction rate will be sensitive to its pK*_a_*. The pH dependence of H_2_O_2_ oxidation kinetics can yield useful information with respect to the mechanism. 

**Figure 1:**
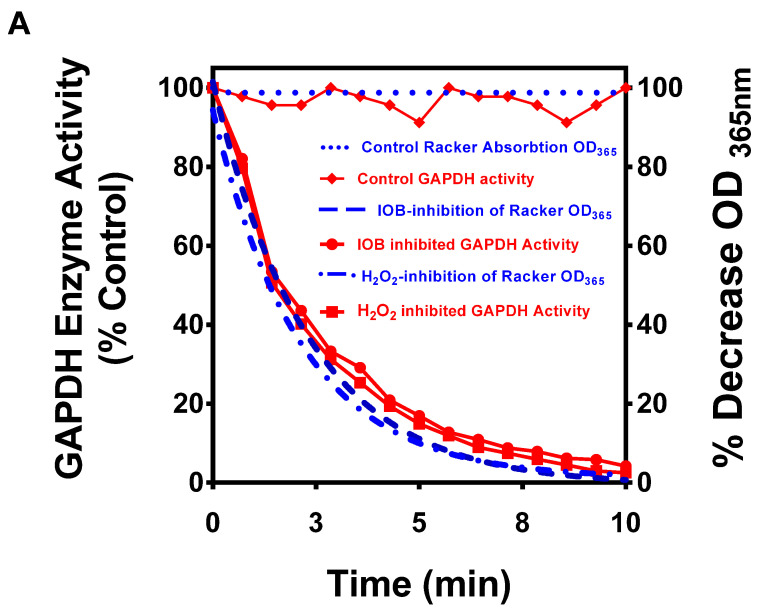

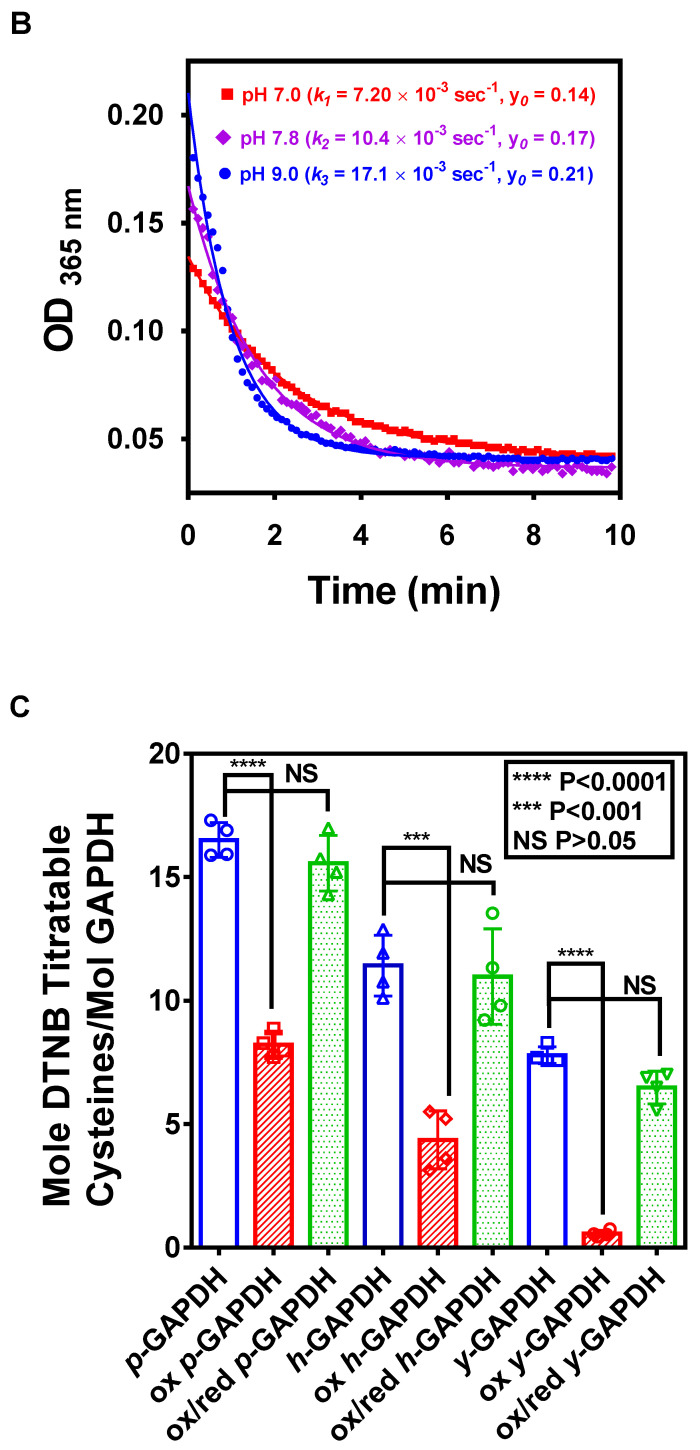

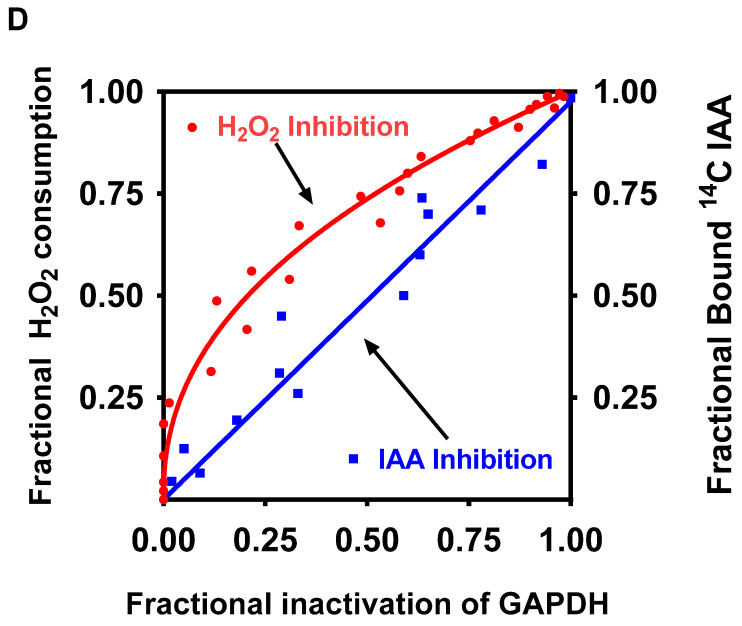
(**A**–**D**) Kinetics and stoichiometry of cysteine residues oxidized by H_2_O_2_. (**A**) Correlation of loss of *p*−GAPDH activity and quenching of Racker absorption by the selective C*_c_*(SH) oxidizing agent IOB and H_2_O_2_ (in the absence of reducing agents) at 14 °C. The similarity of the time−dependence of inactivation by IOB is consistent with H_2_O_2_ oxidation of C*_c_*(SH) to C*_c_*(SOH) inactivating the enzyme. (**B**) pH dependence of initial Racker absorption and rate of Racker absorption quenching is increased by rising pH following the addition of excess H_2_O_2_ to *p*−GAPDH at 14 °C. Fitted rate constants (*k*) and extrapolated initial absorption intensities (y*_0_*) were calculated from mono−exponential decays curve−fits. (**C**) Loss and recovery of DTNB titratable cysteines/mol in native and H_2_O_2_−oxidized *p*, human (*h*)*,* and yeast (*y*)−GAPDH (Mean ± SD, *n* = 4). (**D**) The fractional total consumption of H_2_O_2_ (*y*, left ordinate) and fractional total binding of IAA (*y*, right ordinate) plotted against the fractional inactivation of GAPDH subunit enzyme activity (*x*). The data were fitted to the power function, y*^α^* = x*^β^*. The ratio of exponents determines the stoichiometric ratio of reactants from start to finish of the inactivation process.

End−point titrations of the number of mol H_2_O_2_ consumed oxidizing one mol porcine (*p*)−GAPDH to measure reactant stoichiometry were determined to be 8.1 ± 0.6 (*n* = 3). The kinetics of H_2_O_2_ consumed oxidizing GAPDH at 37 °C were measured independently at pH 7, 7.8, and 9 (reported optimal GAPDH enzyme activity is at pH 8.5[40]), and second−order rate plots were constructed from the kinetic measurements and reactant stoichiometric ratios (Supplementary Figure S1). The kinetics of H_2_O_2_ consumption were monophasic at pH 7 (bimolecular rate constant (*k*) = 9.4 M^−1^s^−1^) and biphasic at higher pH. The rate constant for H_2_O_2_ oxidation of *p*−GAPDH at pH 7 at 37 °C is in good agreement with published data for *r*−GAPDH of 11.4 M^−1^s^−1^ and 10 M^−1^s^−1^ at pH 7.5 at 22 °C [37,41]. This is similar to the rate constant obtained for the H_2_O_2_ oxidation of cysteine [42]. (We discuss these controversial observations in detail in Supplementary Note 1). The resolved rate constants for oxidation reactions that increase (*k*′) and decrease (*k*′′) with rising pH were as follows: pH 7.8, *k*′ = 13.7 M^−1^s^−1^, *k*′′ = 6.6 M^−1^s^−1^; pH 9.0, *k*′ = 25.6 M^−1^s^−1^ and *k*′′ = 2.6 M^−1^s^−1^. Experimental designs to investigate the identity of the pH−dependent biphasic H_2_O_2_ oxidation steps at 37 °C were as follows. The thiol oxidizing agent iodosobenzoic acid (IOB) selectively oxidizes C*_c_*(SH) to sulfenic acid, C*_c_*(SOH) [29] inactivating enzyme activity and quenching the NAD^+^/thiolate [C*_c_*(S^−^)] charge−transfer Racker absorption at 365 nm [43], both process reversed by addition of excess thiol [30,43]. The time−courses of IOB and H_2_O_2_ oxidation of *p*−GAPDH at pH 7 correlated with Racker absorption quenching and loss of enzyme activity assayed in the absence of dithiothreitol (DTT) (Figure 1a). Enzyme activity after incubation with IOB and H_2_O_2_, declined to 2.5 ± 2.8% and 4.2 ± 2.3% control (*n* = 4), respectively. DTT addition restored the activity of the IOB−oxidized enzyme to 95.3 ± 5.7%, in contrast to only 12.9 ± 7.9% with the H_2_O_2_−oxidized *p*−GAPDH.

The pH dependence of *p*−GAPDH C*_c_*(S^−^)−−NAD^+^ Racker absorption was shown to increase with raising the buffer pH [43], as demonstrated in Figure 1b. Measurement of the pseudo−first−order rate constants for Racker absorption quenching after H_2_O_2_ oxidation also increased with increasing the buffer pH, measured at pH 7 at 37 °C (*k_1_*), 7.8 (*k_2_*), and 9 (*k_3_*) (Figure 1b). The ratios of rate constants *k_2_*/*k_1_* = 1.44, and *k***_3_**/*k_1_* = 2.34 correlated with the same ratios of pH−dependence of the bimolecular rate constants *k*′ (*k*′_pH7.8_/*k*′_pH7_ = 1.46, *k*′_pH9_/*k*′_pH7_ = 2.7), but not with *k*′′ (*k*′′_pH7.8_/*k*′′_pH7_ *=* 0.7, *k*′′_pH9_/*k*′′_pH7_ = 0.28). These results establish that Step 1 is the H_2_O_2_ oxidation of C*_c_*(SH) to C*_c_*(SOH), indicating Step 2 is associated with an H_2_O_2_ oxidation step that renders GAPDH refractory to enzyme activity reactivation by excess thiol (DTT).

**Scheme 1:**
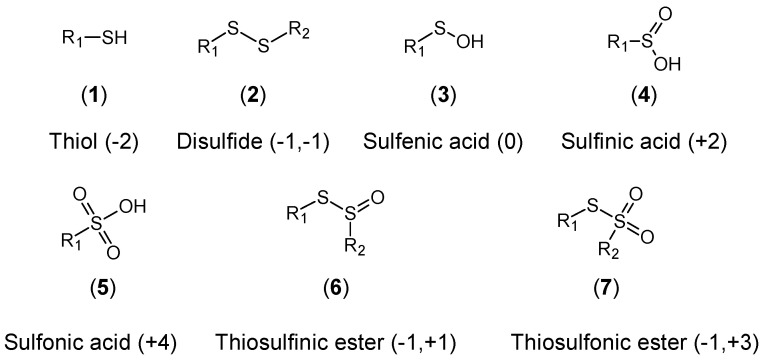
List (1−7) of Common post−translational redox oxidation state values of cysteine residue sulfur atoms found in native cellular proteins and peptides discussed in the manuscript. The sulfur and sulfur − sulfur oxidation states are sown in parenthesis. The list is by no means exhaustive but cover all oxidation states of cysteine discussed in this study.

There were no significant differences noted in the hydrolysis−stable amino acid composition between native and H_2_O_2_−oxidized irreversibly inactivated *p*−GAPDH. Methionine sulfoxide and cysteine sulfonic acid was undetectable in the oxidized sample (Supplementary Table S1). Tryptophane content (measured by fluorescence quantum yield) was also not significantly different between samples of native and H_2_O_2_−oxidized enzymes. The possible oxidation states of cysteine, commonly observed in proteins, are listed in Scheme 1, and all species (including cysteine) are either destroyed or modified by the high−temperature hydrolysis protocol with the exception of cysteine sulfonic acid, which was below the limit of quantitation (BLQ). Modification of cysteines by H_2_O_2_ oxidation of GAPDH by 5,5−dithio−bis−(2−nitrobenzoic acid (DTNB) oxidation of cysteines was determined by 2−nitro−5−thiobenzoic acid (TNB) absorption (ϵ_412_ = 14,150 M^−1^cm^−1^ in *p*−GAPDH, humans (*h*−GAPDH), and yeast (*y*−GAPDH). The primary structure of their subunit homotetramers contains 16, 12, and 8 cysteines/mol GAPDH, respectively (Supplementary Figure S2). After rapid H_2_O_2_ oxidation and denaturation in the DTNB buffer, in four separate experiments, the mean DTT reactive cysteines lost/mol GAPDH from each species were 8.28, 7.05, and 7.23 (Figure 1c), equivalent to 2.07, 1.76, and 1.81 cysteines/subunit, respectively. The maximal TNB absorption following DTNB treatment of the H_2_O_2_−oxidized enzymes diminished for *r*− and *h*−GAPDH over time (see below). DTNB titratable thiol restoration for *p*−, *h*−, and *y*−GAPDH yielded 88.6%, 93.8%, and 81.5% following a cycle of DTT reduction and spin−column buffer exchange (SCBE), respectively (Figure 1c). The reversible loss of both DTNB titratable cysteines/subunit for *y−*GAPDH points to Step 2 as C*_v_*(SH) to C*_v_*(SOH) oxidation.

The premise that both H_2_O_2_ oxidation steps are necessary and sufficient to form irreversibly inactivated GAPDH subunits is verifiable experimentally at pH 7, as described in the Materials and Methods. The fractional consumption of H_2_O_2_ (*y*) as a function of the fractional thiol−irreversible inactivation of *r*−GAPDH (*x*) from initiation to completion of the oxidation process (Figure 1d) and fitted to a power function *y^α′^* = *x ^β′^*, where the exponents *α′* and *β′* are the stoichiometric ratios of the reactants. To irreversibly inactivate one GAPDH subunit (*β′* = 1), the consumption of ~two mol H_2_O_2_ are apparent (*α′* = 2.14 ± 0.13, *r*^2^ = 0.97). When the same analysis is applied to GAPDH inhibition using iodoacetic acid (IAA) to selectively alkylate all four C*_c_*(SH) residues/tetramer abolishing subunit enzyme activity (Figure 1d), the results yielded the established value of *α′* = 1.10 ± 0.07 (*r*^2^ = 0.95) [43]. These results support the premise that both oxidation steps are necessary and sufficient for irreversible inactivation of GAPDH subunit activity.

### 2.2. Identification of Redox−Active Cysteine Intermediates

We established that the targets of H_2_O_2_ oxidation of GAPDH are the two active site cysteine residues. In this section, we explore the sequence of redox steps that result in irreversible enzyme inactivation. Using MS techniques, we determine the identity and oxidation states of the cysteine residues and the degree of homogeneity of these modifications within the four subunits comprising the homotetramer, after achieving redox equilibrium.

**Figure 2:**
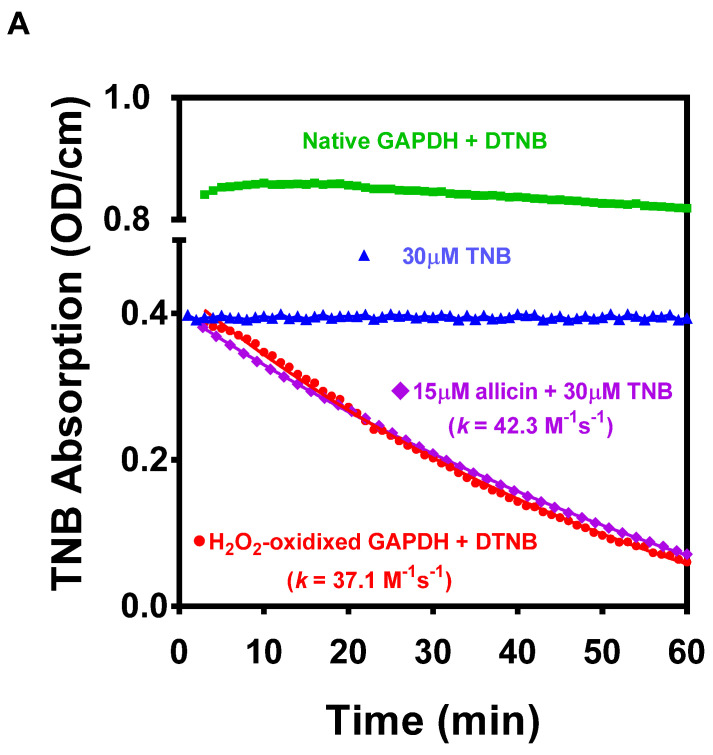

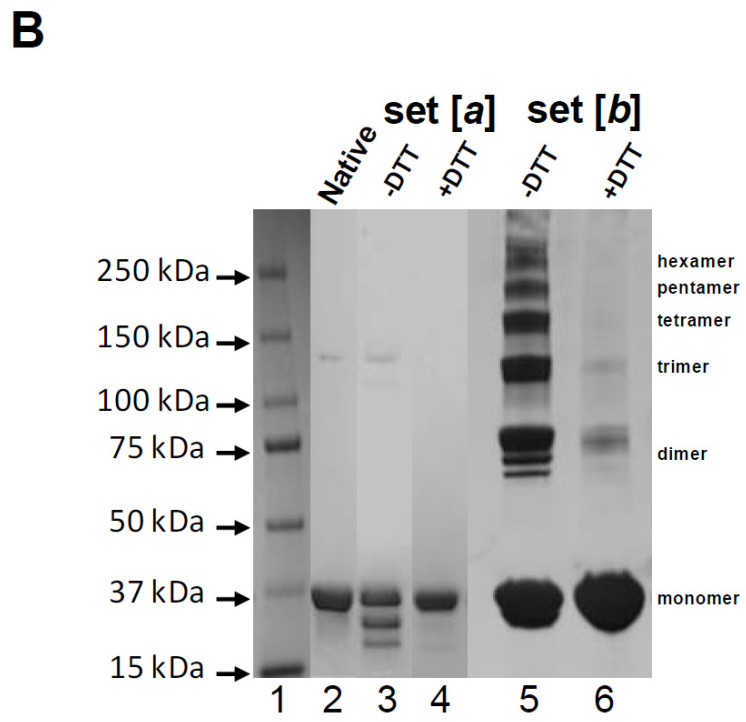
(**A**,**B**) Evidence for thiosulfinic ester and disulfide formation after H_2_O_2_ oxidation of GAPDH. (**A**) Kinetics of TNB absorption of native and H_2_O_2_−oxidized *r*−GAPDH [3.8 nmol/mL] rapidly denatured in the presence of DTNB and 0.1% at RT. TNB released after DTNB oxidation was relatively stable over time (~16 mol TNB/mol GAPDH), as was a control sample of TNB added to the buffer. In contrast, TNB released after DTNB oxidation in H_2_O_2_−oxidized *r*−GAPDH declined over time. Similar kinetics for TNB consumption was observed for admixed TNB and allicin, (2:1 mol/mol). The stoichiometry and kinetic data were used to calculate the bimolecular rate constants for H_2_O_2_−oxidized *r*−GAPDH and allicin demonstrating that the data are consistent with the presence of thiosulfinic esters in the H_2_O_2_−oxidized enzyme. (**B**) The digital SDS−polyacrylamide gel electrophoresis (PAGE) composite figure was constructed by interleaving lanes from different unreduced and reduced gels. (L1) molecular weight markers. (L2) native *r*−GAPDH. (L3, and L4) unreduced and reduced H_2_O_2_−oxidized *r*−GAPDH incubated post−oxidation for 2 h showing the presence of DTT−sensitive gel−shifted banding mobility of >~36 kDa, due to intrasubunit disulfide bonding. (L5 and L6) unreduced and reduced H_2_O_2_−oxidized *r*−GAPDH after denaturation and overnight incubation at pH 8.2, proving the presence of disulfide−linked subunit multimers. The data show evidence for the formation of an intrasubunit disulfide bond formation after incubation of the oxidized enzyme, which equilibrates at mildly alkaline conditions to form DTT−sensitive intrasubunit multimers.

Denaturation of GAPDH following Steps 1 and 2 should afford rapid condensation of sulfenic acids to thiosulfinic esters [44] and be available for reaction with two mol TNB forming two mol mixed disulfide [45]. In the absence of competing nucleophiles, the four thiosulfinic esters/GAPDH tetramer would be expected to stoichiometrically react with the eight remaining cysteines/GAPDH tetramer. Investigation of this premise was conducted by rapid H_2_O_2_ oxidation of *r*−GAPDH and denaturation in DTNB and 0.1% sodium dodecyl sulfate (SDS) buffer at room temperature (RT). Initially, four of the eight DTNB titratable cysteines were lost after oxidation (compared to the native enzyme), (Figure 2a), and as predicted, the liberated TNB was quantitively consumed by the oxidized enzyme. As a control, TNB reaction with the naturally occurring thiosulfinic ester, allicin (S−allyl prop−2−ene−1−sulfinothioate) [45], is also shown in Figure 2a, resulting in a rate constant of 37.1 M^−1^ s^−1^.

Measurement of the bimolecular rate constant for TNB reaction with thiosulfinic ester from TNB absorption requires an accurate determination of the stoichiometry of total bound cysteinylthionitrobenzoate (see Materials and Methods and Supplementary Scheme SI for details). After a total of 3 h additional time of incubation a total of 16.6 ± 0.2 and 15.2 ± 0.3 (*n* = 4) mol TNB/mol were recovered from native and H_2_O_2_−oxidized *r*−GAPDH, respectively. From the data and stoichiometry, a bimolecular rate constant of 42.3 M^−1^ s^−1^ was calculated for TNB reacting with the oxidized enzyme. These results are consistent with a mechanism whereby H_2_O_2_ oxidizes C*_c_*(SH) and C*_v_*(SH) to a thiosulfinic ester in all subunits of the GAPDH homotetramer followed by reaction of the thiosulfinic ester with all inter/intra downstream cysteines forming eight mol disulfides/mol GAPDH.

These results became progressively less reproducible with time if the H_2_O_2_−oxidized enzyme at 14 °C was not immediately denatured but allowed to incubate for a further 5–20 min at 14 °C prior to denaturation in the DTNB buffer. The following experiments were conducted to investigate this discrepancy. Samples of native and H_2_O_2_−oxidized *r*−GAPDH were incubated at 14 °C under N_2_ for 2 h and denatured in 0.1% SDS buffer, and cysteines alkylated with N−ethylmaleimide (NEM). Disulfide content in the denatured alkylated enzymes was measured using 2−nitro−5−thiosulfobenzoate (NTSB) reagent (alkylation and NTSB protocols are described in the Materials and Methods). Disulfide content yielded 0.2 ± 0.1 and 3.7 ± 0.6 (*n* = 4) disulfides/mol native and oxidized *r*−GAPDH, respectively. The same samples (*minus* NEM alkylation) were also probed for cysteine content with DTNB, yielding 16.12 ± 0.60 and 3.88 ± 0.36 cysteines/mol native and oxidized *r*−GAPDH, respectively. Redox modification of the thiosulfinic esters must occur in the H_2_O_2_−oxidized enzyme to explain the recovery of half the expected total number of disulfides.

The relative inter and/or intrasubunit distribution of the ~four mixed disulfides/homotetramer in the post−oxidation incubation was probed by SDS−PAGE gel−shift [46]. Aliquots from native and H_2_O_2_−oxidized samples were either stored at −80 °C [set (*a*)] or adjusted to pH 8.5, incubated overnight under N_2,_ and then stored at −80 °C [set (*b*)]. Native *r*−GAPDH (Figure 2b Lane 1) migrated at ~36 kDa. An unreduced sample from set (*a*) resulted in additional bands with higher mobility than the subunit monomer (Lane 3), collapsing to a single band at ~36 kDa after reduction (Lane 4). This confirms the appearance of ‘gel−shifted’ higher mobility disulfide−linked subunits of GAPDH after long storage [38].

An unreduced sample from set (*b*) migrated as clusters of higher molecular weight bands (Lane 5) that migrated at ~36 kDa after reduction (Lane 6). The mean relative migration of each cluster in Lane 5 was interpolated from standards (Lane 1). Each cluster migrated at unary multiples of GAPDH subunit monomer MW (see Figure 2b right vertical text), confirming the presence of heterogeneous populations of intersubunit disulfide multimers. Moreover, these results indirectly confirm the presence of at least one disulfide and one reduced cysteine in H_2_O_2_−oxidized GAPDH, which forms within the same subunit before denaturation.

We next addressed the conundrum of how a modification to the thiosulfinic ester during incubation can account for these results and attempted to identify the DTNB−unreactive cysteine residue. Electrospray Ionization Quadrupole Time−of−flight/Mass Spectrometry (ESI−QTOF/MS) analysis of native *r*−GAPDH and a reduced sample from set (*a*), yielded major peaks at 35,693 and 35,724.5 Da, respectively (Supplementary Figure S3). There was also a mass increase of 31.5 Da, approximately that of an additional two oxygen atoms/subunit. In order to identify the DTNB−unreactive cysteine noted above, an unreduced sample from set (*a*) was denatured, and all cysteines alkylated with iodoacetic acid (IAA) and incubated at 6 °C for two weeks (to oxidize all thiosulfinic acids to sulfonic acids by dissolved oxygen in the buffer) with a companion sample of native *r*−GAPDH. ESI−QTOF/MS analysis of the native and H_2_O_2_−oxidized and alkylated *r*−GAPDH samples yielded two major peaks. The differences in mass between the sample peaks were +107 and +105 Da. (Supplementary Figure S3). After subtraction of the mass of one carboxymethylated cysteine (+58.05), a mass increase of +49 and +47 Da is consistent with the addition of three oxygen atoms to each subunit.

Next, we used Liquid Chromatography Tandem Mass Spectrometry (LC/MS/MS) to analyze a peptide map of trypsin−digested peptide mapping of a sample of *r*−GAPDH oxidized with H_2_^18^O_2_ followed by simultaneously reduction−alkylation with iodoacetamide−tris(2−carboxyethyl)phosphine) (TCEP−IAM) in 6 M urea followed by SCBE and mailed to Alphalyse for analysis. A search of the Mascot results of the MS/MS peptide fragmentation data of residues 143–159 for alkylated or oxidized modifications at C7, C*_c_*(SH) and C11, C*_v_*(SH), and the database queried for ^16^O and ^18^O isotopic distribution. Results showed that carbamidomethyl cysteine was the only modification at C11, with the conversion of C7 to either cysteine sulfinic acid, C*_c_*(SO_2_H), or sulfonic acid, with C*_c_*(SO_3_H) in approximately equal abundance. The majority (~80%) of each peptide species had one ^18^O atom (Table 1) indicating that (to the nearest integer) one, not two ^18^O atoms in the C*_c_*(SO_2_H)/C*_c_*(SO_3_H) residues derived from H_2_^18^O_2._ Supporting this finding, no peptide species with more than one ^18^O atom were detected. A second significant finding from the data is that sulfenic acid condensation must proceed in a directional manner via nucleophilic attack by C*_c_*(SOH) on C*_v_*(SOH), and not *vice versa*.

In the next step, we used LC/MS for trypsin−digested peptide mass analysis to verify that C*_v_*(SH) was disulfide−bonded and that C*_c_*(SH) was oxidized to C*_c_*(SO_2_H) within the same subunit. We prepared a sample from set (*a*) by alkylation with NEM and SCBE followed by simultaneous alkylation−reduction with IAM−TCEP followed by SCBE and oxidation of all C*_c_*(SO_2_H) residues to C*_c_*(SO_3_H) by HOCl [48] and sent to Alphalyse for analysis. A search of the Mascot analysis of the peptide residues 143–159 containing both (C_7_)C*_c_*(SH) and (C_11_)C*_v_*(SH) revealed three modified peptides. The summed ion−extracted chromatogram (+2, and +3) peak areas showed that IAM alkylated approximately 3.3% of the peptide at both C_7***and***11_, 76.4% modified as C_7***or***11_(SO_2_H) and C_7***or***11_IAM, and 20.3% modified as C_7***or***11_(SO_2_H) and C_11***or***11_NEM. From the MS/MS data in Table 1, where the modification to the sulfinic acid was exclusively determined to be at C_7_, the MS results in Table 1 demonstrate that a small proportion of the peptide is modified to its disulfide [37] and demonstrate that the primary equilibrium H_2_O_2_ oxidation products of *r*−GAPDH subunits are as follows: (1) C*_c_*(SO_2_H); (2) C*_v_*SSC_(*y*_*,_z_*_)_; and (3) C*_y_*,*_z_*(SH), where the subscripts *y* and *z* represent either of the two downstream cysteine residues from C*_v_*(SH).

We conclude from these observations that subunit thiosulfinic esters must undergo further redox modification during the post−oxidation incubation period to be consistent with the experimental data. For example, cysteine thiosulfinic esters undergo hydrolysis at neutral pH (*t*_1/2_ ~15 m) to form forming cysteine thiosulfonic ester as an intermediate [44], a transformation that would be consistent with the experimentally determined products [44].

### 2.3. Steps 1 and 2 Are Exclusively Consecutive

**Scheme 2:**
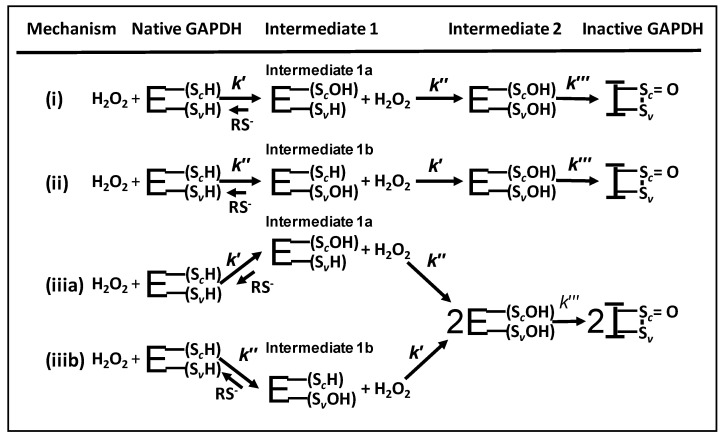
Possible orders for consecutive reaction mechanisms for H_2_O_2_ oxidation of C*_c_*SH and C*_v_*SH. Mechanisms (i) and (ii) occur in exclusive consecutive orders of reaction, resulting in either Intermediate 1a or 1b. Mechanisms (iiia) and (iiib) are randomly consecutive orders of reaction. The ratio of the products of these irreversible consecutive reactions (Intermediates 1a and 1b) are determined by the ratios of k′: k′′. The experimental observations described in the text favor mechanism (i) indicating that H_2_O_2_ oxidation of C*_v_*(SH) is contingent on oxidation of C*_c_*(SH).

There are three possible mechanistic orders (listed in bold roman numerals in Scheme 2) for the order in which subunit H_2_O_2_ oxidation of C*_c_*(SH) and C*_v_*(SH) occurs to yield C*_c_*(SOH) and C*_v_*(SOH): These are either exclusive consecutive (i) C*_c_*(SOH) then C*_v_*(SOH) and (ii) *vice versa*; or two parallel consecutive where either residue is the first residue oxidized (iiia and iiib). We made two observations that lend support for mechanism (i), where the oxidation of C*_c_*(SH) occurs prior to oxidation of C*_c_*(SH). The rate of oxidation of C*_c_*(SH) at pH 9 qualitatively dominates the initial phase of the second−order H_2_O_2_ consumption kinetics (Supplementary Figure S1) and also, the rate increase for the formation of C*_c_*(SOH) with rising pH (Figure 1b) quantitatively mirrors the increase in the measured value of the pH dependence of *k*′ (and opposite to that of *k*′′ as noted above).

### 2.4. In Vitro Evidence That C_v_(SH) Is a Major Factor in Mediating Irreversible GAPDH Activity by H_2_O_2_

A point mutation at C152S in *h*−GAPDH renders the enzyme resistant to H_2_O_2_ oxidative inactivation [34]. We confirmed this finding by comparing H_2_O_2_ oxidation responses of GAPDH from purified cytosolic extracts between two *wt* subspecies of *Lactobacilli*. Some species utilize H_2_O_2_ secretion to control their microflora environment [49]. *L.* a*cidophilus* (an H_2_O_2_−secreting strain where C*_v_*(SH) is replaced by serine) and *L. plantarum* (retaining C*_v_*SH, a non−secretor) [49]. (Table 2 shows the correlation between *Lactobacilli* with C*_v_*S replacement and H_2_O_2_ secretory activity).

**Figure 3:**
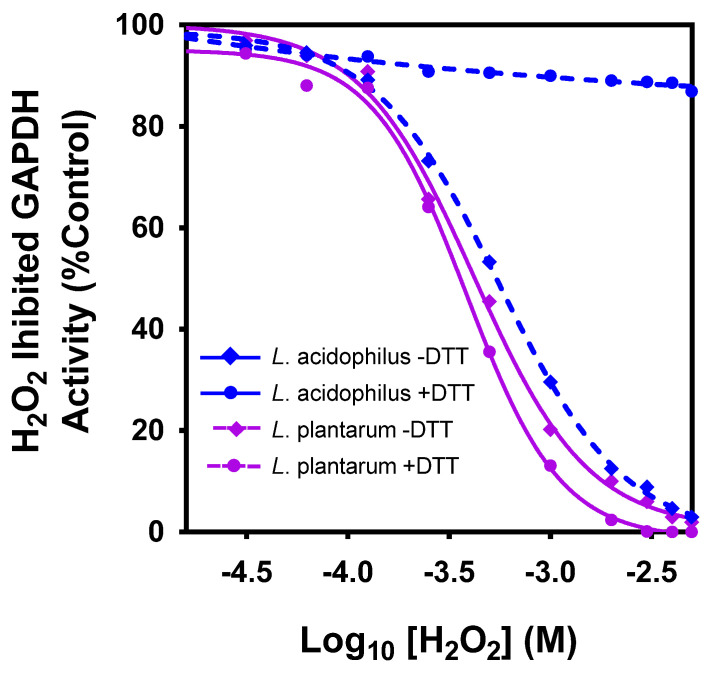
Inactivation of GAPDH in H_2_O_2−_secreting and non−secreting *Lactobacilli***.** Dose–response of H_2_O_2_ inhibition of GAPDH activity in cytosol extracts from *L*. *plantarum*, a non H_2_O_2_ secreting species and *L*. *plantarum*, an H_2_O_2_ secreting species measured in the presence and absence of DTT in the assay buffer. IC_50_ values for H_2_O_2_ inhibition of GAPDH activity calculated from 4−parameter logistic regression analysis of the data The recovery of enzyme activity in the presence of DTT in the species with a C*_v_*S replacement (*L. acidophilus*) supports the premise that C*_v_*(SH) is required for the thiol−irreversible GAPDH inactivation by H_2_O_2_.

The IC_50_ values for H_2_O_2_ inhibition of GAPDH from *L. plantarum* with or without DTT in the assay buffer were 385 and 420 μM (Figure 3), as expected from the DTT−irreversible condensation of C*_c_*(SOH) and C*_v_*(SOH) following H_2_O_2_ oxidation. In contrast, the IC_50_ values for H_2_O_2_ inhibition of GAPDH from *L. acidophilus* assayed in the absence of DTT in the assay buffer were 558.7 μM, but activity was largely restored when DTT was present in the assay buffer, with an estimated irreversible inhibition of ~10% at 5 mM H_2_O_2_. This relatively minor inhibition of *L. acidophilus* GAPDH by H_2_O_2_ is consistent with the sluggish rate (0.4 M^−1^s^−1^) for H_2_O_2_ oxidation of C*_c_*(SOH) to C*_c_*(SO_2_H), as previously observed for C34(SOH) oxidation by H_2_O_2_ to C34(SO_2_H) in human serum albumin [51]. This result adds support to the premise that C*_v_*(SH) is required for thiol−irreversible GAPDH inactivation by H_2_O_2_ and agrees with the isotopic H_2_^18^O_2_ results that the formation of C*_c_*(SO_2_H) is not a major mechanism of GAPDH inactivation by H_2_O_2_.

### 2.5. Subunit Unfolding following H_2_O_2_ Oxidation

In this section, we directly compare subunit unfolding kinetics after the formation of the C*_c_*S(O)SC*_v_* intrachain bond by H_2_O_2_ oxidation of *p*−GAPDH with subunit unfolding kinetics after the formation of the C*_c_*SSC*_v_* intrachain bond after DTNB oxidation of C*_c_*SH. The formation of the thiosulfinic ester should afford identical unfolding kinetics (measured by exposure of a reduced downstream cysteine) as the disulfide, and thiosulfinic ester sulfur–sulfur bonds have similar lengths (~3.1Å) and would provide direct experimental support that the two processes follow mechanistically similar pathways.

In the presence of DTNB [52] formation of the intrasubunit disulfide, C*_c_*(SS)C*_v_* in lobster (*l*−GAPDH) subunits arises from the initial rapid oxidation of C*_c_*(SH) by DTNB (phase 1) forming the mixed disulfide and TNB release. Phase two TNB release results from a nucleophilic attack on the mixed disulfide, forming C*_c_*(SS)C*_v_*, and a second TNB release, followed by phase 3, where subunit unfolding exposes buried C(SH) residues to further reaction with DTNB and TNB release.

This concept is used to measure the pseudo−first−order rate constants (*k_α_*, *kᵦ*, and *k_γ_*) for the three kinetically resolvable DTNB oxidation phases at 14 °C using *p*−GAPDH, calculated from TNB absorption and binding stoichiometry. The established values for the stoichiometric ratios for DTNB oxidization of *l*−GAPDH and TNB release were one each for both phases of TNB release [52] used for the calculation of *k_α_*, and *kᵦ*.

**Figure 4:**
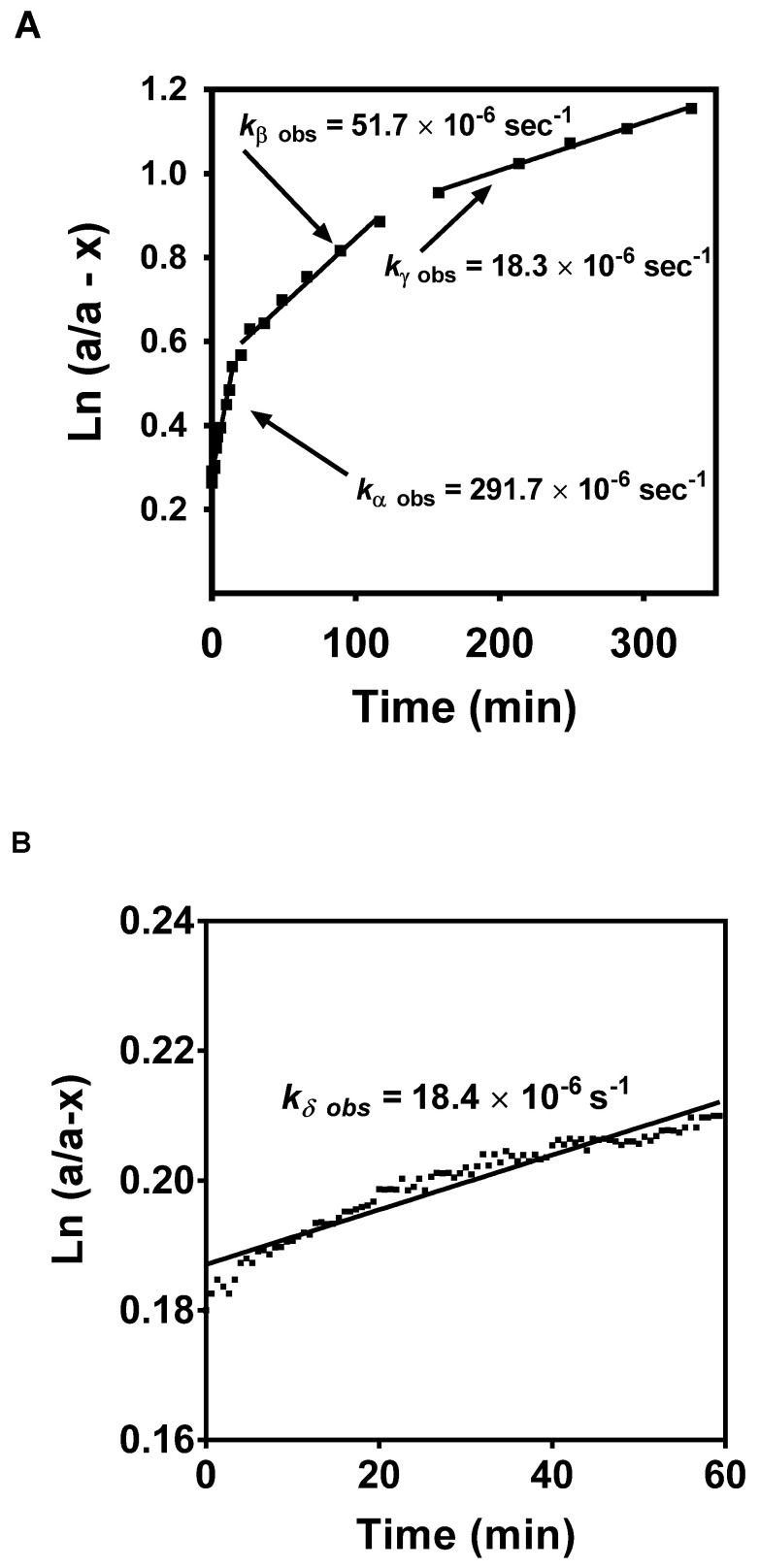

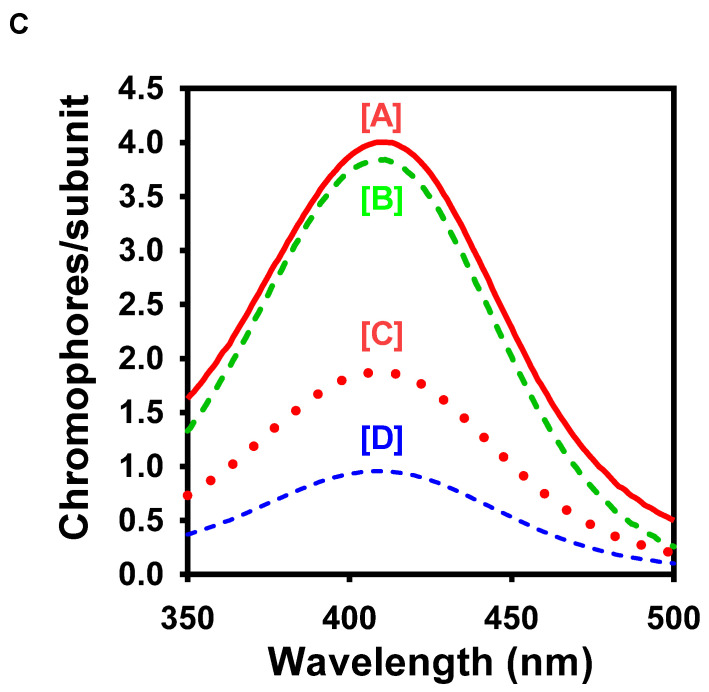
(**A**–**C**) Temporal relationship between GAPDH H_2_O_2_ irreversible inactivation, subunit unfolding, NAD^+^ dissociation, and aggregation: (**A**) Kinetic plots of DTNB reactivity with native *p*−GAPDH at 14 °C by DTNB oxidation of C*_c_*(SH) (*k_α_* **_obs_**), C*_c_*(SS)C*_v_* disulfide formation (*k_β_* _**obs**_), and DTNB oxidation of the two downstream cysteines following subunit unfolding (*k_γ_* _**obs**_). (**B**) Kinetic plot of DTNB reacting with the single cysteine residue exposed after subunit unfolding in H_2_O_2_−oxidized *r*−GAPDH subunits at 14 °C. (**C**) Binding stoichiometry of cysteinylthionitrobenzoate adducts to *r*−GAPDH subunits after incubation with DTNB and SDS−denaturation and SCBE in DTT buffer, as measured by TNB release. [A] TNB release from native *p*−GAPDH. [B] TNB released from SDS−denatured H_2_O_2_−oxidized *r*−GAPDH after a 3 h incubation in the denaturing DTNB buffer (*cf* Figure 1c). [C] TNB released from *p*−GAPDH after completion of the DTNB oxidation kinetic experiment in panel (**A**) for a total extra incubation of 3 h. [D] TNB release after a sample of pre−H_2_O_2_−oxidized *r*−GAPDH after completion of the DTNB oxidation kinetic experiment in panel (**B**) for a total extra incubation of 3 h. Following measurement of the TNB binding stoichiometry, the values of *k_γ_* _**obs**_ and *k_δ_* _**obs**_ could then be calculated.

Measurement of the stoichiometry of bound cysteinylthionitrobenzoate (for calculation of *k_γ._*) is measured after the third phase of DTNB oxidation of *p*−GAPDH is essentially complete. Cysteinylthionitrobenzoate adducts after DTT reduction of a control sample of native denatured *p*−GAPDH denatured in the presence of excess DTNB yielded the expected ~four mol TNB/mol subunit (Figure 4c [A]). The stoichiometry of cysteinylthionitrobenzoate adducts after DTT reduction from a sample of *p*−GAPDH following completion of the third phase (Figure 4c [C]) yielded the expected stoichiometry of ~two mol TNB/mol subunit. The kinetic data and numerical values of rate constants calculated from the absorption and stochiometric data *k_α_*, *k_ᵦ_*_,_ and *k_γ_* are shown in Figure 4a.

The kinetic experiment was repeated using the undenatured H_2_O_2_−oxidized *p*−GAPDH to follow subunit unfolding by exposure of buried cysteines to DTNB (Figure 4b). Experimental controls were first performed to measure the stoichiometries of bound cysteinylthionitrobenzoate to a sample of H_2_O_2_−oxidized *p*−GAPDH rapidly denatured in DTNB buffer and a prolonged incubation to first directly reproduce the data presented in Figure 1c. After SCBE and DTT reduction, ~four mol TNB/mol *p*−GAPDH were recovered (Figure 4c [B] as expected.

Next, the stoichiometry of bound cysteinylthionitrobenzoate was measured after 4 h incubation of the H_2_O_2_−oxidized undenatured enzyme with DTNB after completion of the DTNB oxidation, resulting in the recovery of ~one mol TNB/mol *p*−GAPDH subunit (Figure 4c [D]), enabling calculation of the rate constants (*k_δ_*) of the kinetics of buried cysteine residue exposure (Figure 4b), showing approximate equivalence to *k_γ_* (Figure 4a).

These data support an unfolding mechanism for the H_2_O_2_−oxidized GAPDH subunit that occurs because of conformational strain induced by the formation of an intrachain thiosulfinic ester and re−confirms that only one of the four cysteine residues remains reduced after incubation of the oxidized enzyme.

**Figure 5:**
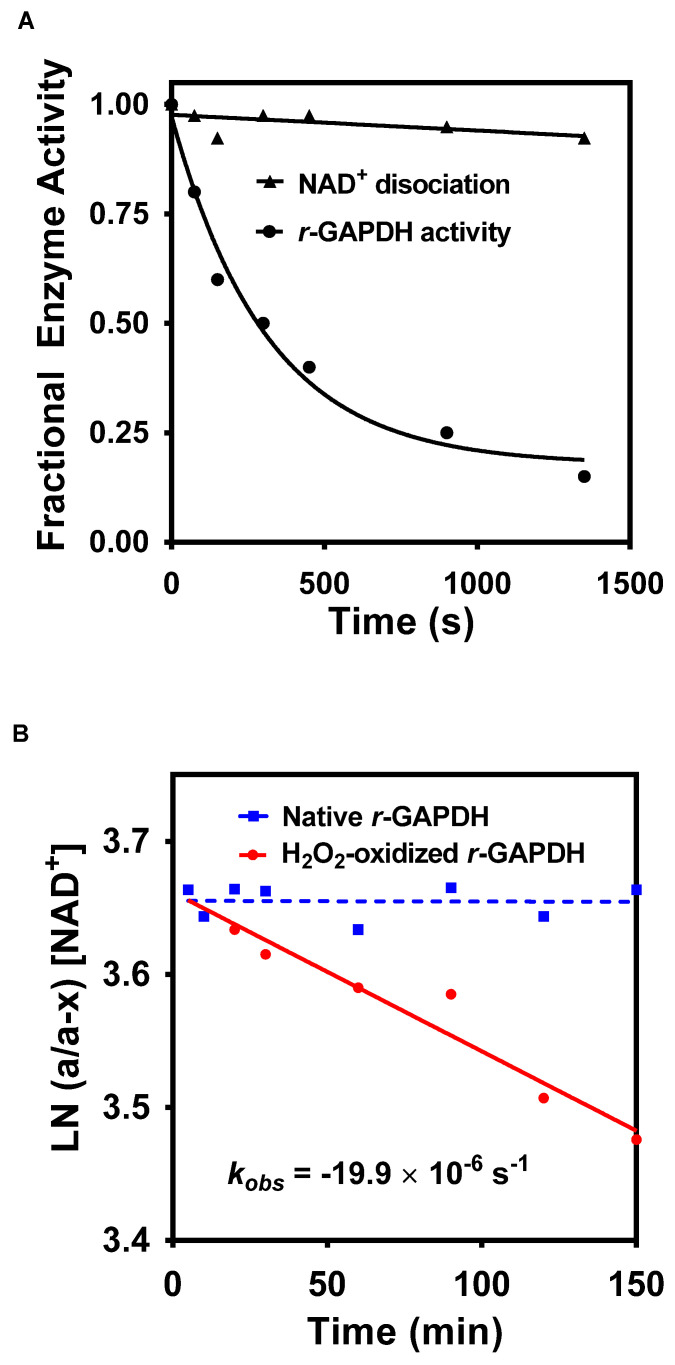

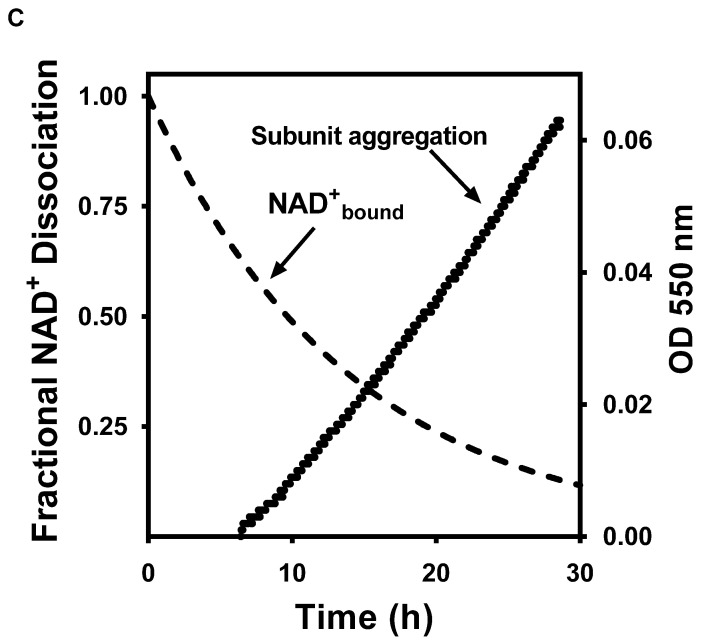
(**A**–**C**) Temporal dissociation between H_2_O_2_−mediated loss of *r*−GAPDH enzyme activity, NAD^+^ dissociation, and subunit aggregation. (**A**) Comparison between time−dependent DTT−irreversible GAPDH activity enzyme activity and dissociation of NAD^+^ from H_2_O_2_−oxidized *r*−GAPDH over 1350 s at 14 °C) (**B**) NAD^+^ dissociation for 150 min at 14 °C in control and H_2_O_2_−oxidized *r*−GAPDH, modeled to a limited first−order protein unfolding process. The similarity of the resulting rate constant to that obtained in Figure 4b indicates that both exposure to buried cysteines and NAD^+^ dissociation arise from subunit unfolding after H_2_O_2_ oxidation. (**C**) *r*−GAPDH aggregation over 30 h at 14 °C. The data show that H_2_O_2_ irreversible GAPDH enzyme inactivation, subunit unfolding, and subunit aggregation are temporally distinct.

The second indicator of subunit unfolding was the measurement of dissociation of bound NAD^+^ from native and H_2_O_2_−oxidized enzymes at 14 °C. Dissociation of NAD^+^ during the timescale for irreversible loss of enzyme activity was relatively minor (Figure 5a), clearly dissociating the kinetics of irreversible enzyme inactivation. NAD^+^ dissociation from H_2_O_2_−oxidized *r*−GAPDH increased over time (Figure 5b). Modeling the data to a generalized limited first−order protein unfolding process, a comparison of the results support a time−dependent exposure of buried cysteines. The data yielded *k_diss_* = 19.95 ± 1.75 × 10^−6^ s^−1^, indicating a temporal connection between subunit unfolding and subunit NAD^+^ dissociation. Subunit aggregation at 14 °C was monitored by forward−angle light scatter (Figure 5c). The data plotted with simulated kinetics of NAD^+^ dissociation over the same time−course show GAPDH subunit aggregation lagged the subunit unfolding process.

### 2.6. H_2_O_2_ Oxidation Perturbs Subunit Structure

**Figure 6:**
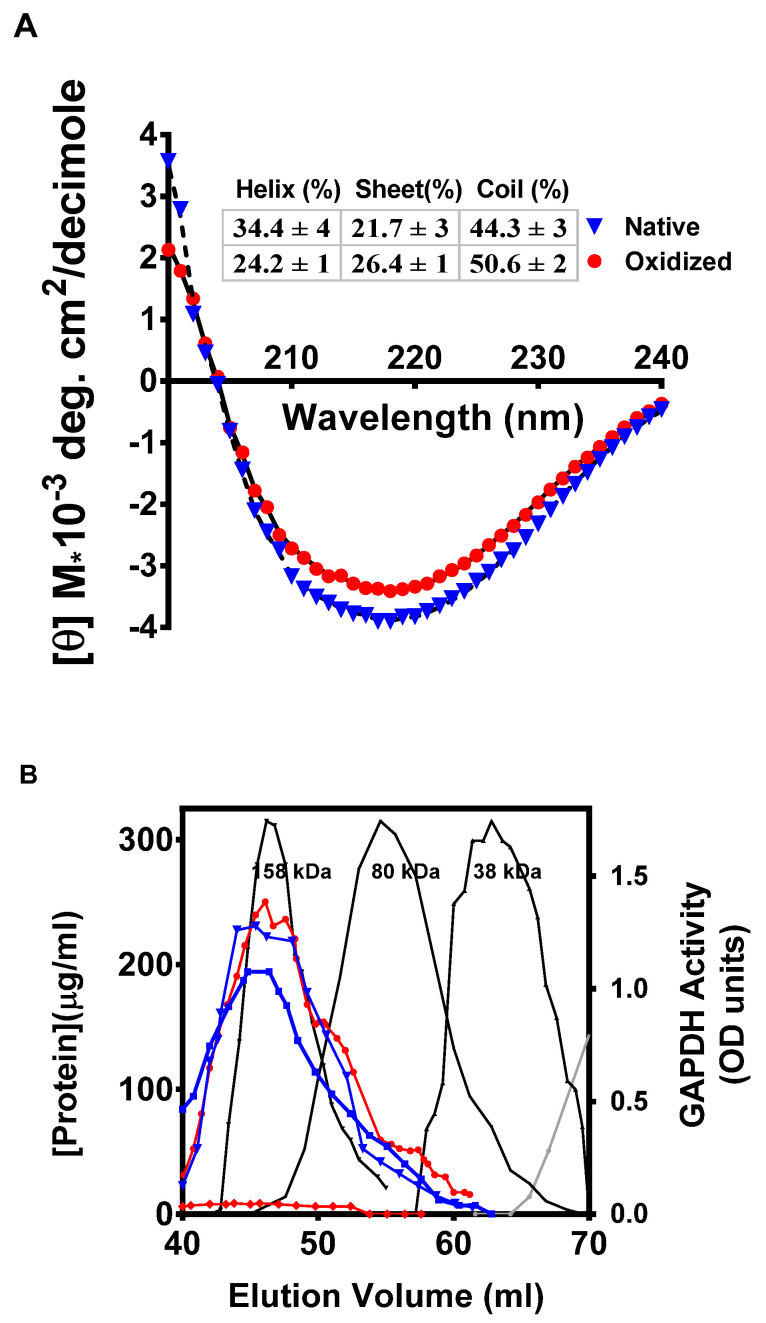
(**A**,**B**)**.** H_2_O_2_ oxidation of GAPDH is accompanied by significant secondary structural changes in the absence of subunit dissociation. (**A**) CD spectra of native and H_2_O_2_−oxidized *p*−GAPDH at 4 °C. Secondary structure parameters from the analysis are tabulated in the insert and show a significant loss of α−helix. The spectra were unchanged between measurement within the shortest timeframe for oxidation and spectra collection and after 1−2 h incubation at 4 °C, showing that the subunit rearrangement was stabilized at low temperature. (**B**) Combined overlaid gel filtration chromatograms of native and oxidized *p*−GAPDH at 4 °C. Calibrator MW standard protein elution profiles are shown in black. Native *p*−GAPDH protein content, native enzyme activity, H_2_O_2_−oxidized *p*−GAPDH protein content, and *p*−GAPDH residual enzyme activity all co−elute from the column at the expected MW of *p*−GAPDH homotetramer at ~148 kDa.

Conformational modifications involving a decrease in α−helical content in H_2_O_2_−oxidized GAPDH using CD spectroscopy were first reported in ref. [38]. Building on this observation, we conducted a more quantitative analysis and determined that the conformation was stable at 4 °C, and its stability was highly temperature−dependent. Analysis of the CD spectra at 4 °C was used to measure conformational changes within the homotetramer secondary structure between native and H_2_O_2_−oxidized *p*−GAPDH, found to be associated with a 34.4% loss of α−helix and an increase in both β−strand (+17.8%) and random coil (+12.5%) (Figure 6a). Loss of α−helical domains is associated with greater local subunit conformational flexibility. The CD spectra measured over two hours showed that the conformation adopted by the oxidized enzyme at low temperature was stable allowing for a realistic timeframe for analysis of its properties.

Having established that the H_2_O_2_−oxidized conformer was stable for 2 h, the influence of H_2_O_2_−oxidized GAPDH on the quaternary structure was probed by gel filtration at 4 °C during this time frame. Chromatograms of native and H_2_O_2_−oxidized *p*−GAPDH resulted in overlapping tetramer elution and enzyme activity profiles (Figure 6b). We previously demonstrated that when GAPDH is inactivated by ~95% by H_2_O_2_, the Michaelis constants for D−glyceraldehyde−3−phosphate (G3P), NAD(H), and P*_i_* are not significantly perturbed [53]. These data show that irreversible enzyme inactivation and subunit secondary structural changes are not associated with either subunit dissociation or interference with adjacent subunit enzyme kinetic parameters.

### 2.7. MD Analysis of C_v_SH Oxidation by H_2_O_2_

The active site environment within the hydrated crystal structure of an isolated subunit *h*−GAPDH [36] was used to construct a van der Waals contact surface diagram demonstrating that in the native enzyme, the C*_v_*(SH) (C156) sulfur atom is located at the back of the ~6Å hydrophilic pocket within the hydrophobic boundary region, accounting for the inability of an H_2_O_2_ molecule within the active site pocket to be able to dock close enough to oxidize C156 in the native enzyme. MDS analysis was applied after C152 was converted to C152(SOH) and H_2_O 440, closest to C156, was replaced by H_2_O_2_ within the Molecular Operating Environment (MOE).

**Figure 7:**
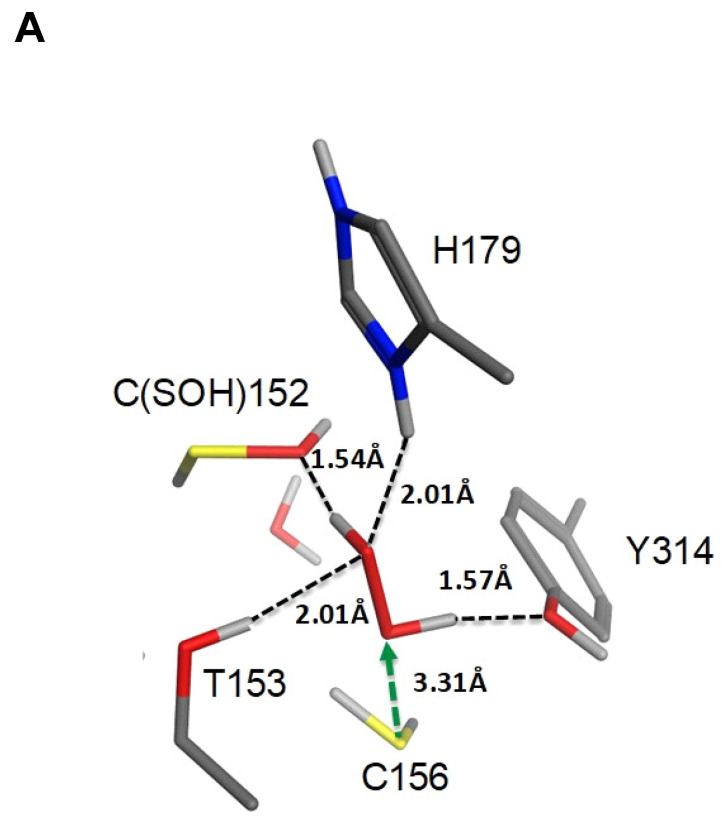

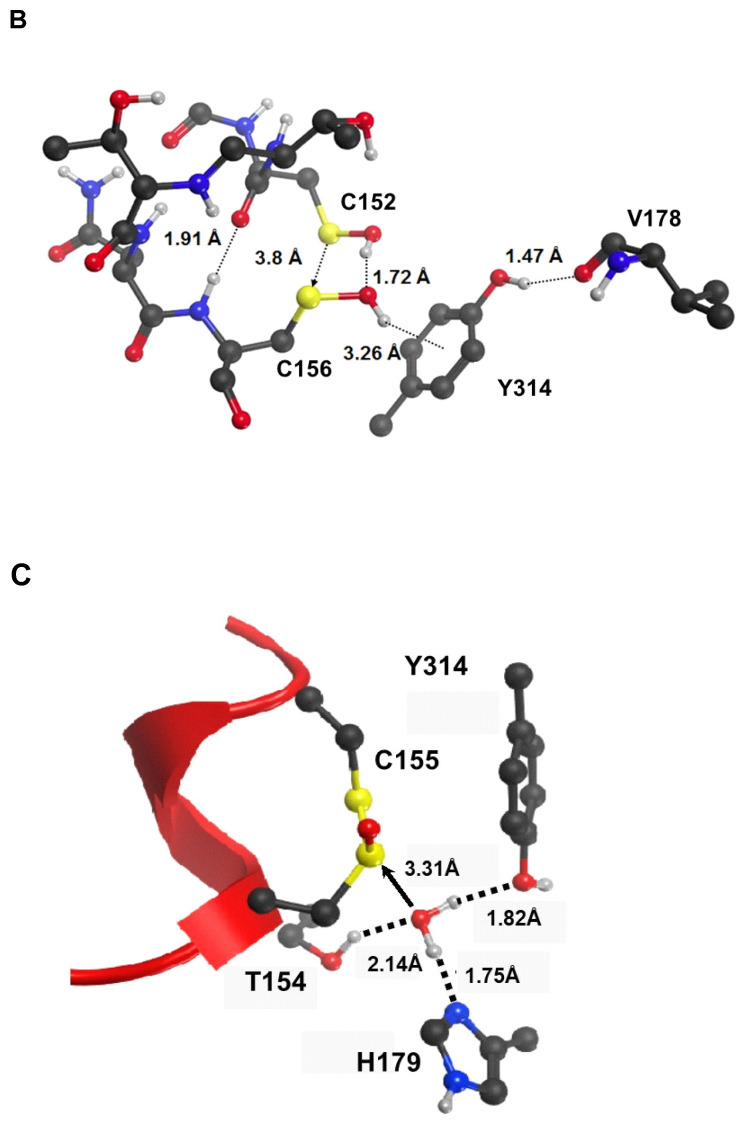
(**A**–**C**)**.** MDS analysis of the environment of C152 and C156 within an isolated *h*−GAPDH subunit to explore the mechanistic interpretation of the biochemical data. (**A**) The catalytic region of *h*−GAPDH (PDB1u8f) within the crystal structure of an isolated subunit shows the placement of an H_2_O_2_ juxtaposed to C156 (C*_v_*(SH). After MDS, the H_2_O_2_ molecule docks forming strong bifurcated hydrogen bond donors (T153, H179) and strong hydrogen bond acceptors (C152 sulfenic acid and Y314) which polarize and activate the H_2_O_2_ molecule for nucleophilic attack by C156. The acidic protons contributed by H179 and C152 sulfenic acid provide a rational basis for the observed decline in H_2_O_2_ oxidation of C156 with rising pH (**B**). Following Steered Molecular Dynamics (SMD), the two sulfur centers are within the van der Waals contact distance for covalent bond formation. Formation of strong H−bonds between the C156 sulfinic acid acidic proton and C152 sulfinate oxygen, as well as the C152 sulfinic acid proton and delocalized Y314 π−orbitals promote nucleophilic attack of its sulfur atom on C156 sulfenic acid sulfur atom as water is an excellent leaving group. The model provides a rational basis for the experimental data proving directional nucleophilic attack resulting in a thiosulfinic acid with the sulfinyl center at C152 and the sulfenyl center at C156. (**C**). A plausible mechanistic reaction pathway for thiosulfonic ester formation is shown depicting a polarizing H−bond donor (T154) and acceptors (H179 and Y314) of a water oxygen atom at the van der Waals covalent sulfur–oxygen bond radius promoting hydroxyl attack on the sulfinyl sulfur center of thiosulfinic ester forming the thiosulfonic ester.

Following energy minimization of the structure (Figure 7a), H_2_O_2_ formed a strong H−bond with the hydroxyl oxygen atom of Y314, the second proton forming a strong H−bond with the oxygen atom of C152(SOH). The H_2_O_2_ oxygen atom furthest from C156 formed an exchangeable H−bond with the proton of the positively charged tautomer of the N_ε2_ imidazole of H179 and an H−bond with the T153 hydroxyl proton. The distance between the oxygen atom of the docked H_2_O_2_ closest to the sulfur atom of C156 was 3.31Å, located at the interface (3.32Å) of their van der Waal’s radii (1.52Å and 1.8Å) for S_N_2 nucleophilic attack. The exchangeable H−bonded proton donated by H178 facilitates heterolytic oxygen bond fission of H_2_O_2_, promoting the water leaving group. The contribution of the acidic protons of both H179 and C*_c_*S(OH) to H_2_O_2_ docking and bond fission provides a rationale for the experimental data demonstrating decreasing reactivity of C*_v_*(SH) with H_2_O_2_ with increasing pH.

### 2.8. MDS Analysis of the Secondary Structure of Oxidized GAPDH

Native subunit sulfur centers of C151 and C156 are separated by 8.6Å, their approach being stabilized by both residues contributing to α−helix and by steric hindrance of the perpendicular Y314 phenol ring [43]. Electrostatic interactions between the sulfur *d2sp3* octahedral electron orbitals and the Y314 π−system also enhance their spatial arrangement. C152(SOH) and C156(SOH) were annotated within MOE, followed by 300 ps MDS, and energy−minimized. The subunit adopted a new stable secondary structure. The first seven residues of the catalytic domain (S151−L157) within the stretch of α−helix are converted to random coil (Supplementary Figure S4). Rearrangement of the H−bonding network was evident: Y314 H−bonds with both C152(SOH) and C156(SOH); H179 H−bonds with T154 and C156(SOH); and T153 H−bonds with T177. α−helix stability is further disrupted by amide H−bond formation between T153 and N155. The conversion of S151−L157 to random coil may directly influence the observed loss of the downstream α−helix (residues 211–221) because of the β−hairpin ‘structural ambivalence’ of this region [54].

Following MDS and energy minimization, the degrees of freedom for the spatial separation of C152(SOH) and C156(SOH) are increased by the loss of α−helix. The aromatic ring axis of Y313 is displaced 2.5Å and tilted 84° to the plane of the path, joining the sulfur centers. Additionally, the random coil sequence in the native subunit residues 224–227 (containing the critical Siah1 binding residue, K227 [55]) forms a 225–226 three−residue turn. Leucine L228 now participates in the 228–234 β−strand. Global secondary structural features showed an overall net loss of 11 α−helix residues (Supplementary Figure S4). The distance separating the C152(SOH) and C156(SOH) sulfur centers is reduced to 7.2Å, just outside the range for sulfenic acid condensation (<5 Å).

### 2.9. Reaction Pathway for Condensation of the Sulfenic Acids

Steered Molecular Dynamics (SMD) [56] was used to explore if the MDS timescale is too short to sample a limiting energy barrier to a closer approach to the sulfur centers. A distance restraint function was applied between the two sulfur atoms of C152(SOH) and C156(SOH) (defined within MOE) within the earlier energy minimized structure with upper and lower boundaries of 4Å and 5Å as the external force. The 100 ps restrained simulation at 300°K was then annealed to 0°K and energy minimized (Figure 7b). The sulfur centers are now 3.8Å which is within the distance for covalent bond formation. The ring axis of Y314 was displaced by 5.6Å and tilted 36.5° to the plane of the path, joining the sulfur centers and removing any steric hindrance of Y314 (Supplementary Figure S5). The secondary structure of the isolated subunit was perturbed with a loss of 29 α−helix, five β−strand, and a gain of 22 random coil residues, in good agreement with CD data for the homotetramer (Figure 5a). This concordance shows that subunit–subunit interactions accommodate the conformational rearrangement.

The local environment of the sulfenic acids in the SMD energy minimized structure was inspected to predict the directional nucleophilic attack by C152(SOH) on C156(SOH) (Figure 7b) to support the validity of the model. A strong H−bond (1.72Å) is present between the hydroxyl proton of C152(SOH) and the hydroxyl oxygen of C156(SOH). The hydroxyl proton of C156(SOH) is polarized by its proximity to the delocalized Y314 π orbital centroid (3.26Å), promoting water as the leaving group. Steric hindrance for the sulfur–sulfur center approach by Y314 is orientationally perturbed by forming a strong H−bond between its hydroxyl proton with the backbone amide carbonyl oxygen of V178 (1.47Å) predicting the experimentally determined directional sulfenic acid condensation for thiosulfinic ester formation.

### 2.10. Pathway for the Formation of a Thiosulfonic Ester

The structure resulting from the earlier simulation was modified within MOE to a thiosulfinic ester, and energy−minimized. A water molecule was placed with its oxygen atom constrained at the van der Waals contact distance (3.31Å) from the partial positively charged thiosulfinic ester sulfinyl center. After local energy minimization, a strong negative polarized transition state of the water oxygen atom is observed (Figure 7c). The oxygen atom of the water molecule forms an H−bond acceptor from the T154 hydroxyl proton. Two water protons form a strong H−bond with the N_δ1_ proton acceptor of histidine H179, and with the Y314 hydroxyl oxygen. The H−bonding network provides a plausible mechanism for orientation and proton abstraction of the water molecule for nucleophilic attack by OH^−^ to form the thiosulfonic ester.

### 2.11. Subunit Instability following Thioester Formation

The SMD structure resulting from the prior simulation was modified within MOE to accommodate the thiosulfonic ester followed by 500 ps MDS at 300°K and energy minimization. The resulting structure revealed a loss of global subunit secondary structure, dissociation of NAD^+^_,_ and solvent accessibility of C244. Complete subunit unfolding in silico was also observed in *h*−GAPDH subunits after C151/C155 disulfide bond formation [34].

We note that destabilization of the monomeric subunit observed after 500 ps of MDS in silico shows that after the formation of the thiosulfinic ester, an intermediary metastable conformation (i.e., the signaling conformer) should in theory be too short−lived to be observable by biochemical and biophysical techniques. This apparent disconnect between the *in silico* result using MDS analysis on the isolated subunit and the homotetrameric CD analysis of the homotetramer potentially arises from the stabilizing influence of the adjacent subunits of the GAPDH homotetramer. It will be of interest to explore this possibility when more advanced MDS computational analytical methods are available for analysis of the H_2_O_2_−oxidized homotetrameric crystal structure.

### 2.12. The Redox−Balanced Reactions of H_2_O_2_ Oxidation of GAPDH

**Scheme 3:**
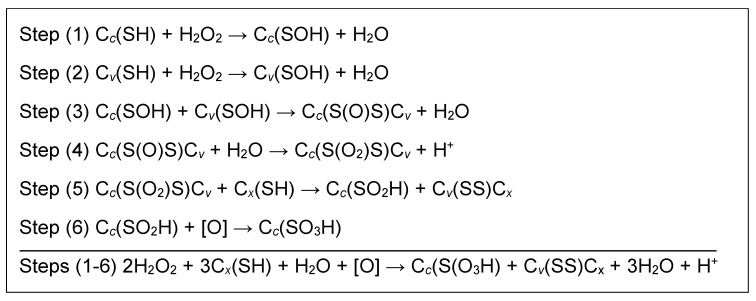
The six individual oxidation steps and overall redox−balanced oxidation equation for the oxidation of *h*−GAPDH by H_2_O_2_.

The overall H_2_O_2_ oxidation of GAPDH involves the three cysteine residues: C*_c_*(SH) (C152); C*_v_*(SH) (C156); and C*_x_*(SH) (C247) in each subunit of the homotetramer. In the case of GAPDH from species with a fourth downstream cysteine residue (porcine or rabbit in this study), the disulfide bond can equilibrate between any pair of intra or intersubunit cysteines other than the oxidized catalytic cysteine sulfinic/sulfonic acids (see Section 2.2). The utility of focusing biochemical studies on the mechanism of GAPDH oxidation by H_2_O_2_ from enzyme species with four rather than three C(SH) residues/subunit is clear from an examination of Scheme 3, as one reduced cysteine is preserved in both *p−* and *r*−GAPDH, providing confidence that sulfur redox equilibrium is reached for proper interpretation of results. Using rapid oxidation and denaturation conditions with excess DTNB, the product of Step (3) is detectable. During an incubation period, the product of Step (4) was predicted from the products found in Step (5). Attempts to investigate the kinetics of Step (4) using biochemical methods were not successful for reasons that may arise from the highly electrophilic and nucleophilic character of thiosulfonic esters [47].

## 3. Discussion

In this study, we uncover key elements of the mechanism by which H_2_O_2_ oxidation of GAPDH irreversibly inactivates enzyme activity, which is coupled with the formation of a metastable conformer that we propose is the major H_2_O_2_ redox signaling species of GAPDH. We demonstrate that the H_2_O_2_ oxidation process of each subunit of the GAPDH homotetramer follows the same mechanistic path resulting in a homogenously oxidized conformer that is stable for at least 2 h at 4 °C. This discovery provides an operable framework for future studies to further explore cognate binding proteins and enzymatic chemical modifications of the putative signaling conformer.

We use MDS, using the crystal structure of an isolated subunit as a tool, to probe the active site hydrogen−bonding network to rationalize and support the biochemical observations while recognizing the limitations of MDS as a surrogate computational model for the homotetramer. However, we note the strong concordance between the calculated loss of α−helix within the oxidized homotetramer by CD in vitro and the computational calculated loss within the isolated subunit in silico. SMD is obviously a ‘forced’ shortcut to overcome computational limitations to demonstrate (but not prove) that a reaction pathway for the observed formation of the intrachain sulfur–sulfur bond is mechanistically feasible.

The conformational rearrangement of inactivated subunits within the homotetramer does not have a major influence on adjacent unoxidized subunit enzyme kinetic measurements [53] or the NAD^+^ co−factor binding (see Section 2.5) at least until the oxidized subunit unfolds. The corollary of this observation is that (prior to subunit unfolding) contacts between adjacent subunits allow for conformational rearrangement of a single oxidized subunit within the homotetramer, which may be sufficient to initiate binding to cognate partner signaling proteins. The ‘induced fit’ model of protein–protein interactions explains how low−affinity interactions with a cognate partner promote conformer selection where protein flexibility is intrinsic to molecular recognition to the detriment of recognition specificity [57]. This concept may apply to the less intrinsically ordered metastable conformation adopted by H_2_O_2_−oxidized GAPDH subunits.

Formation of the thiosulfonic ester occurs during the lifetime of the metastable oxidized subunit, although kinetic analysis of this elusive transformation is beyond the scope of this study. Both thiosulfinate and thiosulfonic esters are reactive electrophiles, although the latter species is also nucleophilic [47]. The existence of a highly electrophilic thiosulfonic ester at the active site of cellular GAPDH oxidized in situ (first proposed by Jeong et al. [33]) may explain the detection of low−abundance modifications of GAPDH in the environment of the crowded chemically diverse cytosol [32,33]. Our data also rationalize that in oxidatively stressed cells, GAPDH can be found as disulfide−linked aggregates with other GAPDH subunits or other proteins, in addition to subunit active site cysteines modified to its sulfinic/sulfonic acid [18,19,34,58,59,60,61].

Given the pivotal role of GAPDH in cell signaling, adequate buffering of the steady−state concentration of a signaling conformer is a critical requirement for mediating cell fate decisions. The consecutive GAPDH two−cysteine switch, applicable to H_2_O_2_ oxidative stress response, has features that meet these expected criteria. In a healthy neuron at physiological H_2_O_2_ levels, the probability of H_2_O_2_ activation of the GAPDH subunit two−cysteine switch would be low [62]. This emphasizes the requirement for a fast response time for initiation of the irreversible signaling conformation once the switch is set. These are precisely the conditions under combined oxidative and metabolic stress (as cytosolic G3P, NAD^+^, glutathione, and ATP levels fall [63,64,65]) which provide an appropriate number of signaling subunits contributing to an appropriately scaled physiological oxidative stress response.

In conclusion, future studies enabling a more complete and accurate characterization of the metastable signaling conformer of GAPDH, with respect to chemical modification and chaperone binding, should facilitate a greater understanding of signaling pathways involved in cell fate decisions, assisting identification of new targets for therapeutic intervention, particularly for chronic neurodegenerative diseases [4,16,18].

## 4. Materials and Methods

### 4.1. GAPDH Preparation

Reagents were purchased from Sigma−Aldrich (St. Louis, MO) unless otherwise indicated. GAPDH from four species: human erythrocyte (*h*−GAPDH), porcine muscle; (*p*−GAPDH) (discontinued), rabbit muscle (*r−*GAPDH), and yeast (*y*−GAPDH). A total of 1–5 mg GAPDH were reconstituted at 1 mg/mL in 1 mM EDTA, 1 mM NAD^+^, 5 mM Na_2_HAsO_4_, pH 7.5, and incubated at 4 °C overnight. Buffer exchange/desalting was accomplished using 7000 MW cutoff Pierce Zeba^TM^ Spin Desalt Columns (Pierce Biotechnology, Rockford, IL, USA), equilibrated according to the manufacturer’s instructions. The sample volume added to the columns, never exceeded the mid−range of recommended sample volume to insure efficient buffer exchange). The method is referred to in the text as Spin Column Buffer Exchange (SCBE). Samples were centrifuged at 14,000× *g* and 0.45 µm filtration, followed by two rounds of spin−column buffer exchange (SCBE) with 50 mM Na_4_P_2_O_7_, 1 mM EDTA, and 1 mM NAD^+^ at the target pH 7. Protein concentration measurements in experimental samples were measured using the Pierce (Rockford, IL, USA) BCA protein assay kit. During this study, *p*−GAPDH was discontinued by Sigma and replaced with *r*−GAPDH, the two homotetramer subunits have 98.8% amino acid sequence homology, and as far as the studies reported in this manuscript, no differences were ever observed between the enzymes regarding H_2_O_2_ oxidation.

### 4.2. GAPDH Activity Assay

GAPDH was assayed as follows [66]. The assay buffer comprised 50 mM Na_4_P_2_O_7_, 1 mM EDTA, 1 mM NAD^+^, 5 mM NaHAsO_4_ containing 50 μg catalase, 0.1% BSA, (S−alkylated with NEM and exhaustively dialyzed) pH 7.5. A 5 mM DTT was included in the assay buffer where indicated. Assays (250 μL) were conducted in 96−well microtiter plates, initiated by addition of 15 μL 50 mM D−glyceraldehyde 3−phosphate. Initial rates of NADH formation were measured at 340 nm (ε = 6220 M^−1^cm^−1^) in a multi−mode BioTek Synergy HT spectrophotometer (Winooski, VT).

### 4.3. H_2_O_2_ Standardization and Assay

2–3% H_2_O_2_ and H_2_^18^O_2_ (90 atom%) were diluted to ~100 mM. The reagents were standardized at 240 nm (ε = 43.6) and diluted to 65 mM and stored in 0.1 mL aliquots at −80 °C. Sample [H_2_O_2_] was measured using the Amplex™ Red H_2_O_2_ assay kit (Invitrogen (Carlsbad, CA, USA) according to the manufacturer’s instructions, resorufin absorption was measured at 570 nm after addition of 20 μL sample for assay.

### 4.4. Measurement of Reduced Cysteines

GAPDH cysteine content was measured using 5,5′−dithiobis−2−nitrobenzoate (DTNB) [67] with modifications. An aliquot of GAPDH was added to the buffer containing 5 mM DTNB, 50 mM Na_4_P_2_O_7_, 0.1% SDS, pH 7.5, and cysteine content measured by TNB absorption (ε_412_ = 14,100 M^−1^cm^−1^). Stoichiometry of Cysteinylthionitrobenzoate (CSSTNB) adducts of GAPDH were determined by removal of TNB and unreacted DTNB by SCBE into 50 mM Na_4_P_2_O_7_, 0.1% SDS, pH 7.5, and protein concentration determined. TNB absorption was measured after addition of 5 mM DTT to reduce CSSTNB, to calculate mol TNB/mol subunit binding stoichiometry.

### 4.5. Oxidation of GAPDH by H_2_O_2_ and Cysteine Titration

The rate of GAPDH oxidation was controlled by varying the concentration of GAPDH and/or H_2_O_2_. Excess unreacted H_2_O_2_ was removed either by SCBE or by addition of 25 µL of washed catalase−conjugated agarose beads/mL (Sigma−Aldrich #C9284), removed by a 10 s pulse spin at 14,000× *g*.

*Rapid oxidation method.* Rapid oxidation was used for all experiments unless indicated otherwise. The reaction buffer contained 2.5 µM GAPDH in 50 mM Na_2_HAsO_4_, 1 mM NAD^+^_,_ pH 7.0. The oxidation was initiated by the addition of 1.5 mM H_2_O_2_ for 10 min at 14 °C. At the end of this period, GAPDH activity both in the presence and absence of 5 mM DTT was reduced to ~95% of native GAPDH activity. Enzyme activity recovered in the presence of DTT is referred to as *reversible* inactivation of GAPDH, while enzyme activity that cannot be recovered in the presence of DTT is referred to as *irreversible* inactivation of GAPDH.

*Post−oxidation incubation conditions.* For determining the status of thiosulfinic ester formation, after rapid oxidation and SCBE, the oxidized enzyme was immediately denatured with 0.1% SDS, followed by addition of DTNB, which immediately reacts with the two unoxidized cysteine residues/subunit, with release of two mol TNB/subunit, and two cysteinylthionitrobenzoate adducts. The two mol TNB then react with the thiosulfinic ester to form a total of four cysteinylthionitrobenzoate adducts.

### 4.6. Demonstration That both Oxidation Steps Irreversibly Inactivate Enzyme Activity

The premise that H_2_O_2_ oxidation Steps 1 and 2 are both necessary and sufficient to form irreversibly inactivated GAPDH subunits is verifiable experimentally.

In the following consecutive equation, two cysteines in each GAPDH subunit (*E*) are oxidized in two steps by H_2_O_2_, followed by a third step resulting in an irreversibly inactivated enzyme (*P*):

$$E+H_{2}O_{2}\;\overset{k^{\prime }}{\to }\;E\left(\mathrm{SOH}\right)+H_{2}O_{2}\overset{k^{\prime \prime }}{\to }E{\left(\mathrm{SOH}\right)}_{2}\;\overset{k^{\prime \prime \prime }}{\to }\;P$$

In the absence of competing reactants (such as thiol reducing agents) where H_2_O_2_ oxidation of cysteine is kinetically irreversible, and in the special case at pH 7 where *k = *$k^{\prime }=k^{\prime \prime }$:

$$E+2H_{2}O_{2}\;\overset{k}{\to }\;E{\left(\mathrm{SOH}\right)}_{2}\;\overset{k^{\prime \prime \prime }}{\to }\;P$$

When *k*‴ > *k*, the consecutive bimolecular reactions above will appear kinetically 3rd order, as the overall reaction rate will depend on the square of the H_2_O_2_ concentration. For a general case, where it is the ratio of stoichiometries of the reactants that is to be determined the rate equation can be expressed in terms of a power law [68], such that the initial reaction rate of formation of *P* (*r*) depends only on the product of the concentrations of reactants raised to the powers of their reaction orders:

*r_0_* = *k*[*P*]*^x^* × [H_2_O_2_]^y^

For elementary reactions, if the reaction goes to completion ([H_2_O_2_] in excess) for the condition *k*‴ > *k*, at any time during the reaction, the reaction orders are equal to their stoichiometric coefficients, and the rate equation for the reaction rate applies throughout the course of the reaction:

*r* = *k*[*P*]^1^ × [H_2_O_2_]^2^

The initial activity of native GAPDH is designated *P*^0^. The reaction is initiated at *t* = 0 by addition of excess H_2_O_2_ at concentration *B^o^* required for completion of GAPDH subunit oxidation. Samples are withdrawn at times *t* during the oxidation process, for (i) determination of the remaining enzyme activity in the presence of excess DTT (*P*′), and (ii) the concentration of H_2_O_2_ remaining in the sample (*B*′). Data are collected over time until *P*′ is reduced to ~5% *P*^o^. From these results, the fractional consumption of H_2_O_2_ (*y*) = (*B^o^*−*B*′) as a function of the corresponding fractional inactivation of subunit activity (*x*) = (*P^o^*−*P*′) modeled to a power function will have the general form *y^α^*′ = *x ^β^*′, where *α*′/*β*′ yields the ratio of the reacting stoichiometries for GAPDH DTT irreversible subunit inactivation by H_2_O_2_. Analysis of the data fit parameters is evaluated for a value of *α*′/*β*′ ~2 demonstrates that *k*‴ > *k* and tests the hypothesis that both oxidation steps are both necessary and sufficient to irreversibly inactivate GAPDH activity.

### 4.7. Detection of Disulfides Using NTSB

Total GAPDH inter−and intrasubunit cystine (CSSC) residues were quantitated using the disodium 2−nitro−5−thiosulfobenzoate (NTSB) reagent described by Thannhauser et al. [69]. H_2_O_2_−oxidized GAPDH after incubation was alkylated with 50 mM NEM at pH 7, and subject to SCBE. A sample was taken for measurement of protein concentration and 25 μL sample was added to the assay solution by dilution of the NTSB solution 1:100 in 3 M guanidine thiocyanate, 0.1 M Na_2_SO_3_, 200 mM Tris, 3 mM EDTA pH 9.5. Alkaline sulfitolysis of one mol disulfide in the presence of NTSB yields one mol TNB, quantitated by absorption at 412 nm, and total mol disulfide/mol subunit determined.

### 4.8. Kinetics of DTNB Reaction with Native and H_2_O_2_−Oxidized GAPDH

DTNB reaction with *l*−GAPDH was described in detail in [52]. DTNB rapidly oxidizes C*_c_*(SH), yielding four mol TNB/mol *l*−GAPDH and four mol C*_c_*(SSTNB). The second phase comprises nucleophilic attack by C*_v_*(S^−^) yielding C*_c_*(SS)C*_v_* and an additional four mol TNB/mol GAPDH, and results in a conformational strain within the subunits, exposing the remainder of the buried subunit cysteines to the solvent, and oxidation by DTNB during the third phase, because of subunit unfolding. The reaction with native and oxidized *r*−GAPDH with DTNB, containing two buried cysteines/subunit in addition to C*_c_*(SH) and C*_v_*(SH) was followed over time by continuous monitoring of the absorption at 412 nm at 14 °C. The three resolvable pseudo−first−order rate constants for each phase were obtained for native *r*−GAPDH using the stoichiometries of 1:1:2 for the three phases of the oxidation. Kinetics of H_2_O_2_−oxidized *r*−GAPDH subunit unfolding, as measured by exposure of buried cysteines were probed by both TNB absorption in the presence of DTNB was observed to assess similarities between subunit unfolding between the native DTNB and H_2_O_2_/DTNB−oxidized enzymes. The rate constant for DTNB reaction with H_2_O_2_−oxidized *r*−GAPDH was calculated from the absorption kinetics and total binding stoichiometry of C(SSTNB).

### 4.9. SDS−PAGE Analysis

Disulfide cross−linked *p−*GAPDH subunits were visualized using SDS−PAGE gel−shift analysis. using Bio−Rad (Hercules CA) Mini−Protean^®^ TGX^™^ pre−cast 10% cross−linked 10−well slab gel cassettes. Samples were diluted with 2 × Laemmli sample buffer. Gels were either run as directed by the manufacturer with samples unreduced or reduced by addition of 10 mM DTT (pH 6.8) and boiling. MW standards were run on each gel (Precision Plus Protein™ Kaleidoscope™ Pre−stained Protein Standards), which were photographed after completion of the electrophoresis. Pinpricks were used to mark the positions of the standards on the gels prior to visualizing using Bio−Rad Silver Stain Plus Kit. Gels were photographed at various development time intervals during silver deposition for selection of optimal development for maximized clarity independently for the unreduced and reduced gels. Digital images from unreduced and reduced lanes were interleaved into one image, with no selective lane digital manipulation.

### 4.10. Thiosulfinic Ester Determination

Cysteine thiosulfinic ester (C(S(=O)S)C) was measured by following the loss of TNB absorption at 412 nm as described for diallylthiosulfinic ester (allicin) [45], where two mol thiol (in this case TNB) react with one mol CyS(=O)Cy to form two mol of the mixed disulfide (CSSTNB). Allicin was prepared exactly as described in ref. [70] and stored in aliquots frozen at −80 °C for single use at 10× working concentration. The stoichiometry of thiol reacting with allicin (2 mol/mol) occurs in two steps the first step is rate−determining [45] shown in the following scheme:


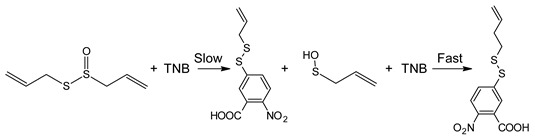


### 4.11. GAPDH Size Exclusion Gel Filtration Chromatography

Native and oxidized GAPDH was chromatographed using 100 mL Sephadex G−100 column, equilibrated at 4 °C with 100 mM NaCl, 50 mM Na_4_P_2_O_7_, 1 mM EDTA. The column was calibrated with 0.4 mL 100 mM NaCl, 50 mM phosphate, 1 mM EDTA containing 13% sucrose buffer with the following standards: Cholesterol oxidase (M*_r_* ~38,000); alkaline phosphatase (M*_r_* ~80,000) and aldolase (M*_r_* ~158,000). The 0.4 mL fractions were collected. After calibration, 3.3 mg native or H_2_O_2_−oxidized samples of *p*−GAPDH were chromatographed, and the eluate fractions were assayed for both GAPDH activity and protein content.

### 4.12. CD Spectroscopy of Native and Oxidized GAPDH

The 200–250 nm CD spectra (1.5 nm bandwidth) on an aliquot of native *p*−GAPDH (0.325 mg/mL in a 0.1 cm cell) was averaged and recorded for 3 s at 0.5 nm intervals in an AVIV 60 DS spectrometer at 4 °C. The CD spectra of oxidized *p*−GAPDH were taken as rapidly as possible after SCBE (equilibrated at 4 °C), and again after incubation in the CD quartz cell for an interval of 1 h at 4 °C. Analysis of the data was performed using CDPro (http://lamar.colostate.edu/~sreeram/CDPro/main.html) after conversion of the CD data to molar ellipticity units) using the SELCON3, CDSSTR, and CONTINLL algorithms to calculate relative α−helix, β−strand, and random coil, expressed as % of total residues. The arithmetic means of the structural results were calculated to remove bias, and the best fit (CDSSTR) is shown for clarity.

### 4.13. Measurement of NAD^+^ Dissociation during H_2_O_2_ Oxidation of GAPDH

0.1 mL samples from GAPDH were concentrated in an Amicon 30 kDa MWCO spin filter and repeatedly centrifuged during the oxidation reaction at 13,000× *g* to generate at least 25 µL of filtrate to determine free [NAD^+^] in the filtrate by HPLC. Total [NAD^+^] and GAPDH activity were determined in a sample taken from the upper chamber. Bound and free NAD^+^ were quantitated by UV absorption of the HPLC eluents using authentic NAD^+^ standards injected onto the column.

### 4.14. Mass Spectrometry Methods

For ESI−QTOF analysis, samples of native and oxidized GAPDH were dissolved in 6:4 CH_3_CN/H_2_O, 0.1% HCOOH. The samples (5 µL, at a flow rate of 10 µL/min) were injected either directly via 50 µm tubing attached to the nebulizing needle or first injected onto an RPLC for MS analysis by ESI−QTOF mass spectrometry (Thermo API−III TQ with an ion−spray interface, Thermo Scientific, Waltham, MA, USA). Data were collected every 3.78 s at a step size of 0.1 Da.

### 4.15. Protein Identification by nano−LC−QTOF Peptide Sequencing and Database Search

*r*−GAPDH samples were analyzed by Alphalyse Inc. (Odens, Denmark). Protein samples were reduced and alkylated with iodoacetamide (IAM) and subsequently digested with trypsin. The resulting peptides were concentrated by lyophilization and re−dissolved for injection on a Dionex nano−LC system and MS/MS analysis on a Bruker Maxis Impact QTOF instrument (Billerica Middlesex County, MA, USA). The identified database protein sequences are shown in the Results together with the obtained mass spectrometric peptide maps. The resulting peptides were analyzed on a nano−LC system connected to a Thermo Orbitrap MS/MS instrument. The MS/MS spectra were used for a Mascot (Matrix Science, Boston, MA, USA) database search against a custom database containing specific protein sequences.

### 4.16. Peptide Mapping with Protein Digestion and LC/MS

Enzymatic digestion. A 5 μg (10 μL) of the GAPDH sample was diluted in 63 μL 50 mM NH_4_HCO_3_ buffer and digested with 4% trypsin (Promega, Madison, WI.) for 3 h at 30 °C. The resulting peptides were desalted by mixed cation exchange and concentrated by Speed Vac lyophilization. The sample was resuspended in 0.1% formic acid and the peptide mapping was performed on a Dionex UltiMate 3000 LC system (Thermo Scientific) coupled to a Bruker Maxis Impact mass spectrometer. The peptides were separated on a ReproSil C18 column (120Å, 3 µm, 15 cm, ID 100 µm, PepSep) using a 30 min gradient with a flow rate of 0.45 μL/min. The MS analysis was performed in positive mode and the peptides were ionized using a Captive Spray source with the following parameters: Capillary 1500 V; NanoBooster 0.2 bar and dry N_2_ gas (3.0 L/min at 150 °C. The mass spectrometer was set for Information Dependent Acquisition in the mass range of 50–2200 m/z. Up to five ions were selected for fragmentation and recorded as MS/MS scans. The selection criteria for fragmentation included a preferred charge state of the precursor ion between +2 and +6. Fragmentation was performed using collision−induced dissociation energy with collision energy ranging from 31–45 eV.

### 4.17. Database Searching and Data Analysis

The raw files were searched through Mascot against an *r*−GAPDH database. The Mascot software finds matching proteins in the database by their peptide masses and peptide fragment masses. For the database search, the search parameters included peptide mass tolerance of 15 ppm and fragment mass tolerance of 0.5 Da. Two missed cleavages were allowed. The peptide identification is based on a probability−scoring algorithm. Only peptides with a mascot score above 20 were used for further data analysis. Data were further manually evaluated in Data Analysis 4.4 (Bruker) and Skyline 20.2.0.343 (MacCoss Lab). For the Skyline analysis, the Mascot search result was used to create a Skyline library and raw data were then imported into the program. Skyline was used to evaluate extracted ion chromatogram traces and isotopic analysis of the peptides.

### 4.18. Preparation of GAPDH Purified from L. plantarum and L. acidophilus

Cell pellets from *L. plantarum* and *L. acidophilus* were grown in 2 L 70142 Lactose broth (Fluka Analytical, Radnor, PA). Cells were lysed in 5 mL of 50 mM Tris pH 8.0, 0.1 mg/mL lysozyme, 1 mM PMSF, 5 mM EDTA followed by sonication and diluted to 45 mL in 0.1% alkylated BSA, 10 mM DTT and centrifuged 30,000× *g*. Supernatants were concentrated to ~0.4 mL in 100 kDa MWCO Centricon^®^ Plus−70 (Millipore, Bedford, MA, USA) units and GAPDH concentrated on 100 mL Sephadex G−100 columns, exactly as previously described, except that 1 mM EDTA, 1 mM NAD^+^ and 50 mNa_4_P_2_O_7_ pH 7 was used to equilibrate the columns. The six 0.4 mL eluting fractions with the highest enzyme activity were pooled (~2.5 mL) and concentrated to 0.5 mL in Centriprep^®^ 30 NMWCO, followed by SCBE. The specific activities of GAPDH in the two semi−purified enzyme preparations were 7.8 and 10.1 μM NAD^+^ reduced/min/mg total protein for *L. plantarum* and *L. acidophilus* GAPDH respectively, the later preparations were diluted to equalize total GAPDH activity.

### 4.19. h−GAPDH Active Site Computational Model Methods

The crystal structure of *h*−GAPDH (PDB1u8f) was used as an active site model. The Molecular Operating Environment (MOE), 2019.01; Chemical Computing Group, 1010 Sherbrooke St. West, Suite #910, Montreal, QC, Canada, was used to construct, solvate, display, and energetically minimize the model. Merck’s Molecular Force Field (MMFF94s with all MOE parameterization was used with a maximum non−bonded cutoff distance of 12.0Å. The GB/VI generalized solvation model with implicit solvent electrostatics [71]. All bound water molecules as determined within the crystal structure were included and immersed in a generated 6Å spherical shell of TIP3 waters. The complex was gently relaxed by tethering the backbone, then minimizing to an rms gradient of 0.05Å. To evaluate if two atomic centers within a protein could potentially approach within a distance for covalent bonding, a modification of MD was employed. The Steered Molecular Dynamics (SMD) method was used [56]. The SMD method imposes an external force in a directional manner (energy) between the atomic centers during MD simulation. In our case distance constraints between atoms were used to provide directionality. This technique allows for the sampling of other conformational states that are separated by higher energy barriers, crossing transitions between equilibrium states that are relatively rare events on the timescale of normal MD timescales (~100 ps), and circumvents the need for long simulation times (~s × 10^−3^) that require supercomputing availability. However, for the nucleophilic attack simulations of peroxide on C152 and C156, the Amber12:EHT force field and parameterization were used because of its overall increased accuracy, particularly for small molecules.

### 4.20. Data Analysis

All curve−fitting and statistical analyses were performed using GraphPad Prism v. 6.04. All data are presented as the mean ($\bar{x}$) and its associated variance, σ (±1 SD). Significance between data sets was determined by ANOVA. For experiment where technical multiple samples (*y*) are used to determine ($\bar{x}$), the result is expressed as$\;\bar{x}\pm S$D, (n = *y*). In experiments where the mean is calculated from the results of *y* separate experiments, the result is expressed as$\;\bar{x}\pm S$D, (N = *y*).

## Figures and Tables

**Table 1:** Mass Spectrometry of modifications to the H_2_O_2_−oxidized tryptic peptide fragment of *r*−GAPDH subunits. (**a**) The active site cysteine residue LC/MS/MS data of (C7, C*_c_*(SH) and vicinal (C11, C*_v_*(SH) “IVSNASCTTNCLAPLAK” inactivated with 90 heavy atom% H_2_^18^O_2_−oxidized *r*−GAPDH detected in the Mascot database search with a Mascot probability score above 19. The table shows the peptide residue mass modifications and associated elution retention times and peak areas. Estimated oxygen isotopic ratios are corrected for the 10% isotopic abundance of ^16^O in the H_2_^18^O_2_ reagent. (**b**) LC/MS approximate estimates of the areas of the extracted ion chromatograms of the active site tryptic peptide from H_2_O_2_−oxidized *r*−GAPDH from sample set [*a*]. The data were searched by Mascot against the *r*−GAPDH sequence for modifications to the charge/mass (m/z) ratios of the 17−mer, which does not distinguish between C7 and C11 modifications. The results indicate that after the first round of alkylation with NEM, ~3% of the tryptic peptide both C*_c_*(SH) and C*_v_*(SH) were unavailable to be alkylated by NEM but readily alkylated with IAM after reduction, indicating the presence of a disulfide bond between the two residues [47]. ~97% of the peptide was modified to cysteine sulfonic acid while ~76% of C*_v_*(SH) was unavailable for modification with NEM. From Table 1a we demonstrate that C7 is exclusively oxidized to its sulfinic acid showing that the majority of (C*_v_*(SH) residue was disulfide−bonded to an intrasubunit downstream cysteine, and unavailable for alkylation with IAM.

**Peptide Sequence**		**Modifying** **Alkylating Agent: IAM**	**R.T. [min]**	**Peak Area**	*Estimated **Isotopic Ratio**
IVSNASC*_c_*[+48]TTNC*_v_*[+57]LAPK		C*_c_*(SO_3_H) C*_v_*(Carbamidomethyl)	15.33	8.79 × 10^7^	17% (^16^O_3_)
IVSNASC*_c_*[+50]TTNC*_v_*[+57]LAPK		C*_c_*(S^16^O_2_^18^OH) C*_v_*(Carbamidomethyl)	15.52	2.7 × 10^8^	83% (^16^O_2_,^18^O)
IVSNASC*_c_*[+32]TTNC*_v_*[+57]LAPK		C*_c_*(SO_2_H) C*_v_*(Carbamidomethyl)	15.18	8.6 × 10^7^	21% (^16^O_2_)
IVSNASC*_c_*[+34]TTNC*_v_*[+57]LAPK		C*_c_*(S^18^O^16^OH) C*_v_*(Carbamidomethyl)	15.4	2.3 × 10^8^	79% (^16^O,^18^O)
**Peptide Sequence**	**Charge**	**Modifying** **Alkylating Agents:** **NEM then IAM**	**R.T.** **[min]**	**Peak Area**	**Summed** **Relative Peak** **Area (%)**
IVSNAS*CTTN*CLAPK	2^+^	*2 × Carbamidomethyl	24.38	5.39 × 10^5^	3.31
IVSNAS*CTTN*CLAPK	3^+^	*2 × Carbamidomethyl	24.38	5.83 × 10^5^	3.31
IVSNAS*CTTN*CLAPK	2^+^	*C(SO_3_H), *C(Carbamidomethyl)	27.29	1.74 × 10^7^	76.42
IVSNAS*CTTN*CLAPK	3^+^	*C(SO_3_H), *C(Carbamidomethyl)	27.29	8.42 × 10^6^	76.42
IVSNAS*CTTN*CLAPK	2^+^	*C(SO_3_H), *C(Ethyl pyrrolidinedione)	30.56	5.07 × 10^6^	20.26
IVSNAS*CTTN*CLAPK	3^+^	*C(SO_3_H), *C(Ethyl pyrrolidinedione)	30.56	1.79 × 10^5^	20.26

**Table 2:** Correlation of GAPDH active site primary sequences of five non−H_2_O_2_ secretors and five H_2_O_2_ secreting *Lactobacilli*, where C*_v_*(SH) is replaced by serine. (Sources: ##Phenotypic data [50]; #sequence data, SIB Bioinformatics Resource.

*Lactobacillus* Species	Accession No.	Active Site	H_2_O_2_ Secretor?
*L. plantarum*	Q88YH6	SCTTN**C**	No
*L. fermentum*	B2GAL7	SCTTS**C**	No
*L. rhamnosus*	C2JVV2	SCTTN**C**	No
*L. brevis*	U2QJ09	SCTTN**C**	No
*L. dulbrueckii*	O32755	SCTTN**S**	Yes
*L. acidophilus*	Q5FL51	SCTTN**S**	Yes
*L. crispatus*	Q5K118	SCTTN**S**	Yes
*L. johnsonii*	C2E5E9	SCTTN**S**	Yes
*L. gasseri*	DIYHE7	SCTTN**S**	Yes

## Data Availability

All data presented in this study is available by written request PAH (Biochemistry) and MOC (MDS).

## Supplementary Materials

The following supporting information can be downloaded at: https://www.mdpi.com/article/10.3390/ijms23094604/s1.

## References

[1] Hyslop P.A., Hinshaw D.B., Scraufstatter I.U., Cochrane C.G., Kunz S., Vosbeck K. (1995). Hydrogen peroxide as a potent bacteriostatic antibiotic: Implications for host defense. Free Radic. Biol. Med..

[2] Hyslop P.A. (1996). Section Review Anti−infectives: Natural mediators of host−defence: The role of H_2_O_2_ in the regulation of bacteriostasis. Expert Opin. Investig. Drugs.

[3] Hyslop P.A., Zhang Z., Pearson D.V., Phebus L.A. (1995). Measurement of striatal H_2_O_2_ by microdialysis following global forebrain ischemia and reperfusion in the rat: Correlation with the cytotoxic potential of H_2_O_2_ in vitro. Brain Res..

[4] Hinshaw D.B., Miller M.T., Omann G.M., Beals T.F., Hyslop P.A. (1993). A cellular model of oxidant−mediated neuronal injury. Brain Res..

[5] Kornberg M.D., Sen N., Hara M.R., Juluri K.R., Nguyen J.V.K., Snowman A.M., Law L., Hester L.D., Snyder S.H. (2010). GAPDH mediates nitrosylation of nuclear proteins. Nat. Cell Biol..

[6] Nakajima H., Amano W., Fujita A., Fukuhara A., Azuma Y.T., Hata F., Inui T., Takeuchi T. (2007). The Active Site Cysteine of the Proapoptotic Protein Glyceraldehyde−3−phosphate Dehydrogenase Is Essential in Oxidative Stress−induced Aggregation and Cell Death. J. Biol. Chem..

[7] Sawa A., Khan A.A., Hester L.D., Snyder S.H. (1997). Glyceraldehyde−3−phosphate dehydrogenase: Nuclear translocation participates in neuronal and nonneuronal cell death. Proc. Natl. Acad. Sci. USA.

[8] Dastoor Z., Dreyer J.L. (2001). Potential role of nuclear translocation of glyceraldehyde−3−phosphate dehydrogenase in apoptosis and oxidative stress. J. Cell Sci..

[9] Sen N., Hara M.R., Kornberg M.D., Cascio M.B., Bae B.-I., Shahani N., Thomas B., Dawson T.M., Dawson V.L., Snyder S.H. (2008). Nitric oxide−induced nuclear GAPDH activates p300/CBP and mediates apoptosis. Nat. Cell Biol..

[10] Hildebrandt T., Knuesting J., Berndt C., Morgan B., Scheibe R. (2015). Cytosolic thiol switches regulating basic cellular functions: GAPDH as an information hub?. Biol. Chem..

[11] Lazarev V.F., Guzhova I.V., Margulis B.A. (2020). Glyceraldehyde−3−phosphate Dehydrogenase is a Multifaceted Therapeutic Target. Pharmaceutics.

[12] Tossounian M.A., Zhang B., Gout I. (2020). The Writers, Readers, and Erasers in Redox Regulation of GAPDH. Antioxidants.

[13] Butera G., Mullappilly N., Masetto F., Palmieri M., Scupoli M.T., Pacchiana R., Donadelli M. (2019). Regulation of Autophagy by Nuclear GAPDH and Its Aggregates in Cancer and Neurodegenerative Disorders. Int. J. Mol. Sci..

[14] Kim J.H., Lee S., Park J.B., Lee S.D., Kim J.H., Ha S.H., Hasumi K., Endo A., Suh P.G., Ryu S.H. (2003). Hydrogen peroxide induces association between glyceraldehyde 3−phosphate dehydrogenase and phospholipase D2 to facilitate phospholipase D2 activation in PC12 cells. J. Neurochem..

[15] Sirover M.A. (2021). The role of posttranslational modification in moonlighting glyceraldehyde−3−phosphate dehydrogenase structure and function. Amino Acids.

[16] Butterfield D.A., Hardas S.S., Lange M.L. (2010). Oxidatively modified glyceraldehyde−3−phosphate dehydrogenase (GAPDH) and Alzheimer’s disease: Many pathways to neurodegeneration. J. Alzheimer’s Dis..

[17] Sirover M.A. (2020). Moonlighting glyceraldehyde−3−phosphate dehydrogenase: Posttranslational modification, protein and nucleic acid interactions in normal cells and in human pathology. Crit. Rev. Biochem. Mol. Biol..

[18] Gerszon J., Rodacka A. (2018). Oxidatively modified glyceraldehyde−3−phosphate dehydrogenase in neurodegenerative processes and the role of low molecular weight compounds in counteracting its aggregation and nuclear translocation. Ageing Res. Rev..

[19] Semenyuk P., Barinova K., Muronetz V. (2019). Glycation of alpha−synuclein amplifies the binding with glyceraldehyde−3−phosphate dehydrogenase. Int. J. Biol. Macromol..

[20] Delport A., Kins S., Hewer R. (2020). The amyloid precursor protein affects glyceraldehyde 3−phosphate dehydrogenase levels, organelle localisation and thermal stability. Mol. Biol. Rep..

[21] Sekar S., Taghibiglou C. (2020). Nuclear accumulation of GAPDH, GluA2 and p53 in post−mortem substantia nigral region of patients with Parkinson’s disease. Neurosci. Lett..

[22] Ping Z., Fan H., Wen C., Ji Z., Liang S. (2021). GAPDH siRNA Regulates SH−SY5Y Cell Apoptosis Induced by Exogenous alpha−Synuclein Protein. Neuroscience.

[23] Tsai C.W., Tsai C.F., Lin K.H., Chen W.J., Lin M.S., Hsieh C.C., Lin C.C. (2020). An investigation of the correlation between the S−glutathionylated GAPDH levels in blood and Alzheimer’s disease progression. PLoS ONE.

[24] Gui H., Gong Q., Jiang J., Liu M., Li H. (2021). Identification of the Hub Genes in Alzheimer’s Disease. Comput. Math. Methods Med..

[25] Hara M.R., Snyder S.H. (2006). Nitric Oxide−GAPDH−Siah: A Novel Cell Death Cascade. Cell. Mol. Neurobiol..

[26] Li C., Feng J.J., Wu Y.P., Zhang G.Y. (2012). Cerebral ischemia−reperfusion induces GAPDH S−nitrosylation and nuclear translocation. Biochemistry.

[27] Nakajima H., Itakura M., Kubo T., Kaneshige A., Harada N., Izawa T., Azuma Y.T., Kuwamura M., Yamaji R., Takeuchi T. (2017). Glyceraldehyde−3−phosphate Dehydrogenase (GAPDH) Aggregation Causes Mitochondrial Dysfunction during Oxidative Stress−induced Cell Death. J. Biol. Chem..

[28] Muronetz V.I., Medvedeva M.V., Sevostyanova I.A., Schmalhausen E.V. (2021). Modification of Glyceraldehyde−3−Phosphate Dehydrogenase with Nitric Oxide: Role in Signal Transduction and Development of Apoptosis. Biomolecules.

[29] Parker D.J., Allison W.S. (1969). The mechanism of inactivation of glyceraldehyde 3−phosphate dehydrogenase by tetrathionate, o−iodosobenzoate, and iodine monochloride. J. Biol. Chem..

[30] You K.S., Benitez L.V., McConachie W.A., Allison W.S. (1975). The conversion of glyceraldehyde−3−phosphate dehydrogenase to an acylphosphatase by trinitroglycerin and inactivation of this activity by azide and ascorbate. Biochim. Biophys. Acta.

[31] Woo H.A., Rhee S.G. (2010). Immunoblot detection of proteins that contain cysteine sulfinic or sulfonic acids with antibodies specific for hyperoxidized cysteine−containing sequences. Methods Redox Signal..

[32] Hwang N.R., Yim S.H., Kim Y.M., Jeong J., Song E.J., Lee Y., Lee J.H., Choi S., Lee K.J. (2009). Oxidative modifications of glyceraldehyde−3−phosphate dehydrogenase play a key role in its multiple cellular functions. Biochem. J..

[33] Jeong J., Jung Y., Na S., Jeong J., Lee E., Kim M.S., Choi S., Shin D.H., Paek E., Lee H.Y. (2011). Novel oxidative modifications in redox−active cysteine residues. Mol. Cell. Proteom..

[34] Peralta D., Bronowska A.K., Morgan B., Doka E., Van Laer K., Nagy P., Grater F., Dick T.P. (2015). A proton relay enhances H2O2 sensitivity of GAPDH to facilitate metabolic adaptation. Nat. Chem. Biol..

[35] Lia A., Dowle A., Taylor C., Santino A., Roversi P. (2020). Partial catalytic Cys oxidation of human GAPDH to Cys−sulfonic acid. Wellcome Open Res..

[36] Jenkins J.L., Tanner J.J. (2006). High−resolution structure of human D−glyceraldehyde−3−phosphate dehydrogenase. Acta Crystallogr. Sect. D Biol. Crystallogr..

[37] Barinova K.V., Serebryakova M.V., Eldarov M.A., Kulikova A.A., Mitkevich V.A., Muronetz V.I., Schmalhausen E.V. (2020). S−glutathionylation of human glyceraldehyde−3−phosphate dehydrogenase and possible role of Cys152−Cys156 disulfide bridge in the active site of the protein. Biochim. Biophys. Acta Gen. Subj..

[38] Vaidyanathan V.V., Sastry P.S., Ramasarma T. (1993). Regulation of the activity of glyceraldehyde 3−phosphate dehydrogenase by glutathione and H_2_O_2_. Mol. Cell. Biochem..

[39] Azam S., Jouvet N., Jilani A., Vongsamphanh R., Yang X., Yang S., Ramotar D. (2008). Human Glyceraldehyde−3−phosphate Dehydrogenase Plays a Direct Role in Reactivating Oxidized Forms of the DNA Repair Enzyme APE1. J. Biol. Chem..

[40] Mountassif D., Baibai T., Fourrat L., Moutaouakkil A., Iddar A., El Kebbaj M.S., Soukri A. (2009). Immunoaffinity purification and characterization of glyceraldehyde−3−phosphate dehydrogenase from human erythrocytes. Acta Biochim. Biophys. Sin..

[41] Elkina Y.L., Kuravsky M.L., El’darov M.A., Stogov S.V., Muronetz V.I., Schmalhausen E.V. (2010). Recombinant human sperm−specific glyceraldehyde−3−phosphate dehydrogenase: Structural basis for enhanced stability. Biochim. Biophys. Acta.

[42] Barton J.P., Packer J.E., Sims R.J. (1973). Kinetics of the reaction of hydrogen peroxide with cysteine and cysteamine. J. Chem. Soc. Perkin Trans. II.

[43] Harris J.I., Waters M., Boyer P.D. (1976). Glyceraldehyde−3−phosphate dehydrogenase. The Enzymes.

[44] Nagy P., Ashby M.T. (2007). Reactive sulfur species: Kinetics and mechanism of the hydrolysis of cysteine thiosulfinate ester. Chem. Res. Toxicol..

[45] Miron T., Rabinkov A., Mirelman D., Weiner L., Wilchek M. (1998). A spectrophotometric assay for allicin and alliinase (Alliin lyase) activity: Reaction of 2−nitro−5−thiobenzoate with thiosulfinates. Anal. Biochem..

[46] Rath A., Glibowicka M., Nadeau V.G., Chen G., Deber C.M. (2009). Detergent binding explains anomalous SDS−PAGE migration of membrane proteins. Proc. Natl. Acad. Sci. USA.

[47] Mampuys P., McElroy C.R., Clark J.H., Orru R.V.A., Maes B.U.W. (2019). Thiosulfonates as Emerging Reactants: Synthesis and Applications. Adv. Synth. Catal..

[48] Hamann M., Zhang T., Hendrich S., Thomas J.A. (2002). Quantitation of protein sulfinic and sulfonic acid, irreversibly oxidized protein cysteine sites in cellular proteins. Methods Enzymol..

[49] Strus M., Brzychczy−Wloch M., Kochan P., Heczko P. (2004). Hydrogen peroxide produced by *Lactobacillus* species as a regulatory molecule for vaginal microflora. Med. Doświadczalna Mikrobiol..

[50] Strus M., Brzychczy−Wloch M., Gosiewski T., Kochan P., Heczko P.B. (2006). The in vitro effect of hydrogen peroxide on vaginal microbial communities. FEMS Immunol. Med. Microbiol..

[51] Turell L., Botti H., Carballal S., Ferrer−Sueta G., Souza J.M., Duran R., Freeman B.A., Radi R., Alvarez B. (2008). Reactivity of sulfenic acid in human serum albumin. Biochemistry.

[52] Wassarman P.M., Major J.P. (1969). The reactivity of the sulfhydryl groups of lobster muscle glyceraldehyde 3−phosphate dehydrogenase. Biochemistry.

[53] Hyslop P.A., Hinshaw D.B., Halsey W.A., Schraufstatter I.U., Sauerheber R.D., Spragg R.G., Jackson J.H., Cochrane C.G. (1988). Mechanisms of oxidant−mediated cell injury. The glycolytic and mitochondrial pathways of ADP phosphorylation are major intracellular targets inactivated by hydrogen peroxide. J. Biol. Chem..

[54] Patel S., Balaji P.V., Sasidhar Y.U. (2007). The sequence TGAAKAVALVL from glyceraldehyde−3−phosphate dehydrogenase displays structural ambivalence and interconverts between alpha−helical and beta−hairpin conformations mediated by collapsed conformational states. J. Pept. Sci..

[55] Bae B.I., Hara M.R., Cascio M.B., Wellington C.L., Hayden M.R., Ross C.A., Ha H.C., Li X.J., Snyder S.H., Sawa A. (2006). Mutant Huntingtin: Nuclear translocation and cytotoxicity mediated by GAPDH. Proc. Natl. Acad. Sci. USA.

[56] Isralewitz B., Gao M., Schulten K. (2001). Steered molecular dynamics and mechanical functions of proteins. Curr. Opin. Struct. Biol..

[57] Janin J., Sternberg M.J. (2013). Protein flexibility, not disorder, is intrinsic to molecular recognition. F1000 Biol. Rep..

[58] El Kadmiri N., Slassi I., El Moutawakil B., Nadifi S., Tadevosyan A., Hachem A., Soukri A. (2014). Glyceraldehyde−3−phosphate dehydrogenase (GAPDH) and Alzheimer’s disease. Pathol. Biol..

[59] Lazarev V.F., Nikotina A.D., Semenyuk P.I., Evstafyeva D.B., Mikhaylova E.R., Muronetz V.I., Shevtsov M.A., Tolkacheva A.V., Dobrodumov A.V., Shavarda A.L. (2016). Small molecules preventing GAPDH aggregation are therapeutically applicable in cell and rat models of oxidative stress. Free Radic. Biol. Med..

[60] Zaffagnini M., Marchand C.H., Malferrari M., Murail S., Bonacchi S., Genovese D., Montalti M., Venturoli G., Falini G., Baaden M. (2019). Glutathionylation primes soluble glyceraldehyde−3−phosphate dehydrogenase for late collapse into insoluble aggregates. Proc. Natl. Acad. Sci. USA.

[61] Lazarev V.F., Tsolaki M., Mikhaylova E.R., Benken K.A., Shevtsov M.A., Nikotina A.D., Lechpammer M., Mitkevich V.A., Makarov A.A., Moskalev A.A. (2021). Extracellular GAPDH Promotes Alzheimer Disease Progression by Enhancing Amyloid−beta Aggregation and Cytotoxicity. Aging Dis..

[62] Cyrne L., Antunes F., Sousa−Lopes A., Diaz−Berrio J., Marinho H.S. (2010). Glyceraldehyde−3−phosphate dehydrogenase is largely unresponsive to low regulatory levels of hydrogen peroxide in Saccharomyces cerevisiae. BMC Biochem..

[63] Schraufstatter I.U., Hyslop P.A., Hinshaw D.B., Spragg R.G., Sklar L.A., Cochrane C.G. (1986). Hydrogen peroxide−induced injury of cells and its prevention by inhibitors of poly(ADP−ribose) polymerase. Proc. Natl. Acad. Sci. USA.

[64] Barinova K.V., Serebryakova M.V., Muronetz V.I., Schmalhausen E.V. (2017). S−glutathionylation of glyceraldehyde−3−phosphate dehydrogenase induces formation of C150−C154 intrasubunit disulfide bond in the active site of the enzyme. Biochim. Biophys. Acta.

[65] Spragg R.G., Hinshaw D.B., Hyslop P.A., Schraufstatter I.U., Cochrane C.G. (1985). Alterations in adenosine triphosphate and energy charge in cultured endothelial and P388D1 cells after oxidant injury. J. Clin. Investig..

[66] Velick S.F., Hayes J.E. (1953). Phosphate binding and the glyceraldehyde−3−phosphate dehydrogenase reaction. J. Biol. Chem..

[67] Ellman G.L. (1959). Tissue sulfhydryl groups. Arch Biochem. Biophys..

[68] Atkins P., Atkins P.W., de Paula J. (2006). The rates of chemical reactions. Atkins’ Physical Chemistry.

[69] Thannhauser T.W., Konishi Y., Scheraga H.A. (1984). Sensitive quantitative analysis of disulfide bonds in polypeptides and proteins. Anal. Biochem..

[70] Lawson L.D., Wood S.G., Hughes B.G. (1991). HPLC analysis of allicin and other thiosulfinates in garlic clove homogenates. Planta Med..

[71] Labute P. (2008). The generalized Born/volume integral implicit solvent model: Estimation of the free energy of hydration using London dispersion instead of atomic surface area. J. Comput. Chem..

