# Supplementary material for: Mechanism of GAPDH Redox Signaling by H2O2 Activation of a Two−Cysteine Switch
Source: Int J Mol Sci. 2022 Apr 21;23(9):4604. doi: 10.3390/ijms23094604 (PMC9102624; doi:10.3390/ijms23094604)
Supplement: Supplementary file 1 [file ijms-23-04604-s001.zip › ijms-1659129-supplementary.pdf]

Supplementary Information Section

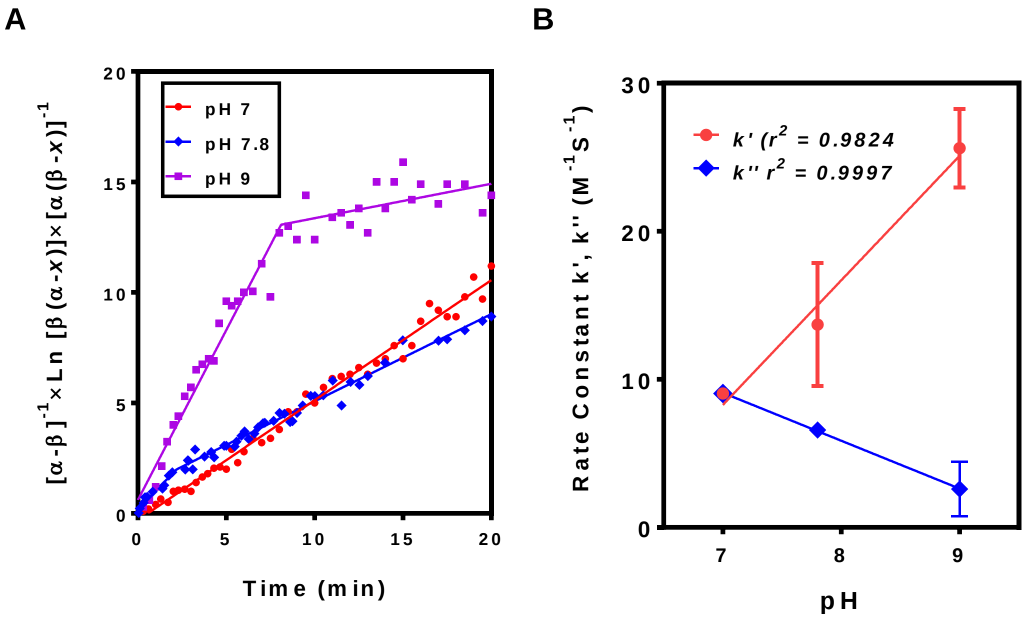

Monomodal Rate Constant Modelling pH 7		Bimodal Rate Constant Modelling pH 7.8		Bimodal Rate Constant Modelling pH 9	
**Method: linear regression (No Constraints)**		**Method Segmental linear regression (No Constraints)**		**Method Segmental linear regression (No Constraints)**	
Slope: (*k* * 10^3 M^{-1} min^{-1})	0.5432 ± 0.009013	Slope 1: (*k'* * 10^3 M^{-1} min^{-1})	0.8222	Slope 1: (*k '* * 10^3 M^{-1} min^{-1})	1.537
		Slope 2: (*k''* * 10^3 M^{-1} min^{-1})	0.3945	Slope 2: (*k''* * 10^3 M^{-1} min^{-1})	0.156
		**Std. Error**		**Std. Error**	
		slope1	0.1235	Slope 1	0.07871
		slope2	0.00846	Slope 2	0.05449
**95% Confidence Interval**		**95% Confidence Interval**		**95% Confidence Interval**	
Slope	0.5250 to 0.5613	slope1	0.5738 to 1.071	slope 1	1.377 to 1.696
P value	< 0.0001	slope2	0.3775 to 0.4115	slope 2	0.04553 to 0.2662
Degrees of Freedom	44	Degrees of Freedom	48	Degrees of Freedom	38
R square	0.988	R square	0.9876	R square	0.9699
		Absolute Sum of Squares	3.274	Absolute Sum of Squares	25.87
Sy.x	0.3638	Sy.x	0.2612	Sy.x	0.8252

Supplementary Figure S1. Related to 'Stoichiometry and pH-dependent kinetics of GAPDH oxidation' Regression plots and statistics. (**a**) Segmental linear regression plots of H_2O_2 oxidizing *p*-GAPDH with accompanying statistical analysis of the data, and calculation of bimolecular rate constants for H_2O_2 reaction with *p*-GAPDH using initial molarity of H_2O_2 (α) and GAPDH subunit H_2O_2 oxidizable cysteine residues (β), and reactant stoichiometries. The latter was determined from end-point measurements to be (for one mol GAPDH) ~eight mol H_2O_2/eight mol cysteine residues (*cf* **Figure 1c**). Measurement of decrease in $[H_2O_2]$ over time yields (*x*) the molar amounts of GAPDH subunit cysteines oxidized by H_2O_2 at time *t*. The rate equation for this process is given by $\frac{dx}{dt}=k(\alpha-x)(\beta-x)$, after integration and rearrangement yields $kt=[\alpha-\beta]^{-1}\times\ln[\beta(\alpha-x)]\times[\alpha(\beta-x)]^{-1}$. The data was plotted against time to determine the rate constant (*k*) from the slope of the linear regression analysis. For biphasic reaction kinetics, the values of the resolved rate constants were calculated by bimodal segmental linear regression analysis with GraphPad Prism 6. The mono-modal rate constant *k'*, pH 7 or bimodal rate constants, *k', k''*, pH 7.8 and 9 (in units of $M^{-1}min^{-1}$) was estimated from the slopes of the plots by segmental linear regression analysis using GraphPad Prism 6. Statistical metrics from the analysis are shown in the table below**.** (**b**) Linear regression analysis of plots of the resulting rate constants for *k'', k''*($M^{-1}s^{-1}$) with associated 95% C.I., were plotted as a function of buffer pH. The fitted data demonstrate that GAPDH oxidation by H_2O_2 comprises two kinetically distinguishable reaction steps, which increase (*k'*) and decrease (*k''*) as the pH of the buffer is raised.

		Native *p*-GAPDH		Oxidized *p*-GAPDH	
**Amino Acid**	**Residues per mole subunit mole subunit**	**µmol/ml**	**Calculated residues per mole subunit**	**µmol/ml**	**Calculated residues per mole subunit**
**ASX**	38	3.0013	40.25	2.4976	40.14
**THR**	22	1.6212	21.74	1.3568	21.81
**SER**	19	1.2755	17.11	1.0682	17.17
**GLX**	18	1.5146	20.31	1.2492	20.08
**PRO**	12	0.9843	13.2	0.8427	13.54
**GLY**	32	2.5619	34.36	2.1327	34.28
**ALA**	32	2.4465	32.81	2.044	32.85
**VAL**	34	2.4185	32.43	2.0036	32.2
**MET**	9	0.6189	8.3	0.501	8.05
**ILE**	21	1.4285	19.16	1.207	19.4
**LEU**	18	1.4488	19.43	1.216	19.54
**TYR**	9	0.7614	10.21	0.6151	9.89
**PHE**	14	1.118	14.99	0.9277	14.91
**HIS**	11	0.7993	10.72	0.6713	10.79
**LYS**	26	2.0081	26.93	1.6814	27.02
**ARG**	10	0.7427	9.96	0.6154	9.89
***TRY**	**3**	**-**	**3**	**-**	**3**
**CYS(SO_3H)**	-	BLQ	BLQ	BLQ	BLQ
**MET(S=O)**	-	BLQ	BLQ	BLQ	BLQ
***Calculated by fluorescence quantum yield of denatured enzyme**					

Supplementary Table S1. Related to 'Stoichiometry and pH-dependent kinetics of GAPDH oxidation.' Lack of primary structure amino acid modification (except cysteine) by H_2O_2 in *p*-GAPDH subunits the acid hydrolysis of protein samples destroys cysteines and sulfinic acids, but not cysteine sulfonic acids, which in addition to methionine sulfoxide, were not detected in the samples. Tryptophan is also destroyed by acid hydrolysis and was quantitated by comparison of tryptophan fluorescence quantum yield (before hydrolysis) from a denatured sample of H_2O_2-oxidized *p*-GAPDH and normalized to the fluorescence quantum yield of a denatured native sample of *p*-GAPDH with three tryptophan residues in the primary subunit structure.

```
P04406|Human         MGKVKVGVNGFGRIGRLVTRAAFN----SGKVDIVAINDPFIDLNYMVYMFQYDSTHGKF 56
P00355|Pig           --MVKVGVNGFGRIGRLVTRAAFN----SGKVDIVAINDPFIDLHYMVYMFQYDSTHGKF 54
P46406|Rabbit        --MVKVGVNGFGRIGRLVTRAAFN----SGKVDVVAINDPFIDLHYMVYMFQYDSTHGKF 54
Q88YH6|L.plantarum   -MSVKIGINGFGRIGRLAFRRILELGEKSSDIEVVAINDLT-SPALLAHLLKYDSTHGTL 58
Q5FL51|L.acidophilus -MTVKIGINGFGRIGRLAFRRIMDLGEKSKDIEVVAINDLT-TPALLAHLLKYDSTHGTF 58
P00359|Yeast         --MVRVAINGFGRIGRLVMRIALS----RPNVEVVALNDPFITNDYAAYMFKYDSTHGRY 54

P04406|HUMAN         HGTVKAENGKLVINGNPITIFQERDPSKIKWGD-AGAEYVVESTGVFTTMEKAGAHLQGG 115
P00355|PIG           HGTVKAENGKLVINGKAITIFQERDPANIKWGD-AGATYVVESTGVFTTMEKAGAHLKGG 113
P46406|RABBIT        HGTVKAENGKLVINGKAITIFQERDPANIKWGD-AGAEYVVESTGVFTTMEKAGAHLKGG 113
Q88YH6|L.plantarum   NADVSATDDSIVVNGKNYRVYAEPQAQNIPWVKNDGVDFVLECTGFYTSKAKSQAHLDAG 118
Q5FL51|L.acidopilus  DHEVSSTEDSIVVDGKKYRVYAEPQAQNIPWVKNDGVDFVLECTGFYTSKAKSQAHLDAG 118
P00359|Yeast         AGEVSHDDKHIIVDGKKIATYQERDPANLPWGS-SNVDIAIDSTGVFKELDTAQKHIDAG 113
                                                                    152 156
                                                                     |   |
P04406|Human         AKRVIISAPSAD-APMFVMGVNHEKYDNSLKIISNASCTTNCLAPLAKVIHDNFGIVEGL 174
P00355|Pig           AKRVIISAPSAD-APMFVMGVNHEKYDNSLKIVSNASCTTNCLAPLAKVIHDHFGIVEGL 172
P46406|Rabbit        AKRVIISAPSAD-APMFVMGVNHEKYDNSLKIVSNASCTTNCLAPLAKVIHDHFGIVEGL 172
Q88YH6|L.Plantarum   AKRVLISAPAGSDLKTIVYNVNDDILTADDRIVSAGSCTTNCLAPLAFFENKEFGIKVGT 178
Q5FL51|L.Acidophilus VKRVLISAPAGNDLKTIVYSVNQDTLTADDKIVSAGSCTTNSLAPMVNALQKEFGIEVGT 178
P00359|Yeast         AKKVVITAPSST-APMFVMGVNEEKYTSDLKIVSNASCTTNCLAPLAKVINDAFGIEEGL 174
                        179
                         |
P04406|Human         MTTVHAITATQKTVDGPS-GKLWRDGRGALQNIIPASTGAAKAVGKVIPELNGKLTGMAF 233
P00355|Pig           MTTVHAITATQKTVDGPS-GKLWRDGRGAAQNIIPASTGAAKAVGKVIPELNGKLTGMAF 231
P46406|Rabbit        MTTVHAITATQKTVDGPS-GKLWRDGRGAAQNIIPASTGAAKAVGKVIPELNGKLTGMAF 231
Q88YH6|L.plantarum   MTTIHAYTSTQMLLDGPVRGGNFRAARAAGVNTIPHSTGAAKALGLVIPELNGKLQGHAQ 238
Q5FL51|L.acidophilus MTTIHAYTSTQMILDGPVRGGNLRAARAAAINIIPHSTGAAKAIGLVIPELNGKLNGHAQ 238
P00359|Yeast         MTTVHSLTATQKTVDGPS-HKDWRGGRTASGNIIPSSTGAAKAVGKVLPELQGKLTGMAF 231

P04406|Human         RVPTANVSVVDLTCRLEKPAKYDDIKKVVKQASEGPLKGILGYTEHQVVSSDFNSDTHSS 293
P00355|Pig           RVPTPNVSVVDLTCRLEKPAKYDDIKKVVKQASEGPLKGILGYTEDQVVSCDFNSDTHSS 294
P46406|Rabbit        RVPTPNVSVVDLTCRLEKAAKYDDIKKVVKQASEGPLKGILGYTEDQVVSCDFNSATHSS 295
Q88YH6|L.plantarum   RVGVVDGSLTELVAILDKKVTADEVNAAIKKHTEGN--ESFGYNDDEIVSSDVIGTTFGS 296
Q5FL51|L.acidophilus RVPVPDGSVTELVSILGKNVTADEVNEAMKKYE--S--PSFEYEPNNVVSSDILGRTAGS 294
P00359|Yeast         RVPTVDVSVVDLTVKLNKETTYDEIKKVVKAAAEGKLKGVLGYTEDAVVSSDFLGDSHSS 291
                                                   314
                                                    |
P04406|Human         TFDAGAGIAL---NDHFVKLISWYDNEFGYSNRVVDLMAHMASKE 335
P00355|Pig           TFDAGAGIAL---NDHFVKLISWYDNEFGYSNRVVDLMVHMASKE 333
P46406|Rabbit        TFDAGAGIAL---NDHFVKLISWYDNEFGYSNRVVDLMVHMASKE 333
Q88YH6|L.plantarum   IFDPTQTEVTSDGDNQLVKTVAWYDNEYGFTCQMVRTLLKFATL- 340
Q5FL51|L.acidophilus IFDPTQTMVTTAGDKQLVKTVAWYDNEYSFTCQMVRTLLHFATL- 338
P00359|Yeast         IFDASAGIQL---SPKFVKLVSWYDNEYGYSTRVVDLVEHVAKA- 332
```

Supplementary Figure S2. Related to ‘Stoichiometry and pH-dependent kinetics of GAPDH oxidation.’ Sequence Alignments of GAPDH subunits from distinct species**.** Alignments were calculated using Swiss Prot Bioinformatics (Sievers, F, *et al*. ‘*Fast, scalable generation of high-quality protein multiple sequence alignments using Clustal Omega*.’ Molecular systems biology. 2011;7:539). Annotated residue number start with the initiating methionine (as used in the sequence of *h*-GAPDH PDB1u8f). Red residues: trypsin cleaved peptides that have cysteine residues in *h*, *p, r*-GAPDH. C_c(SH) and C_v(SH) in *h*-GAPDH are annotated with the conventional crystallographic annotation, where residues are numbered to include the initiating methionine. Purple residues: Active site sequence of from two *wt* subspecies of *Lactobacilli L. plantarum*, a non-H_2O_2 secretor, and *L. acidophilus*, an H_2O_2 secretor[50]. Yeast GAPDH has only two cysteines/subunit, C_c(SH) and C_v(SH).

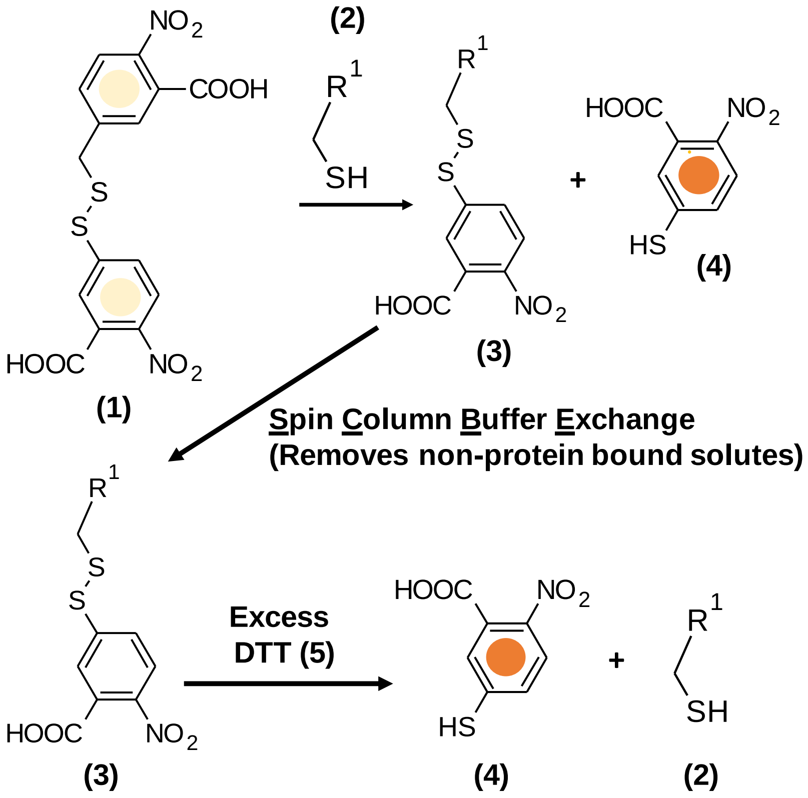

(1) 5,5-dithio-bis-(2-nitrobenzoate) (DTNB)

(2) Protein cysteine residues

(3) Cysteinylthionitrobenzoate [C(SSTNB]

(4) 2-Nitro-5-thiobenzoate (TNB, ϵ (412nm) = 14,150 $M^{-1}cm^{-1}$)

(5) Dithiothreitol (DTT)

Supplementary Scheme S1. Related to 'Identification of redox-active cysteine intermediates. Spectrophotometric measurement of protein cysteine content and protein cysteinyl-TNB adducts. The TNB absorption spectra were measured between 350-500 nm. DTNB oxidizes cysteine residues to generate TNB, and its absorption at 412 nm is proportional to cysteine content. SCBE (separation of DTNB and TNB the incubation from protein cysteinylthionitrobenzoate adducts) after denaturation in 0.1% SDS buffer followed by DTT reduction liberates the bound TNB is quantitated from its absorption spectra and molar extinction coefficient at 412nm . This last step is essential in this work for ensuring that a full accounting of DTNB oxidation of proteins and subsequent reaction of TNB with higher oxidation states of cysteine is applied to interpret the experimental results.

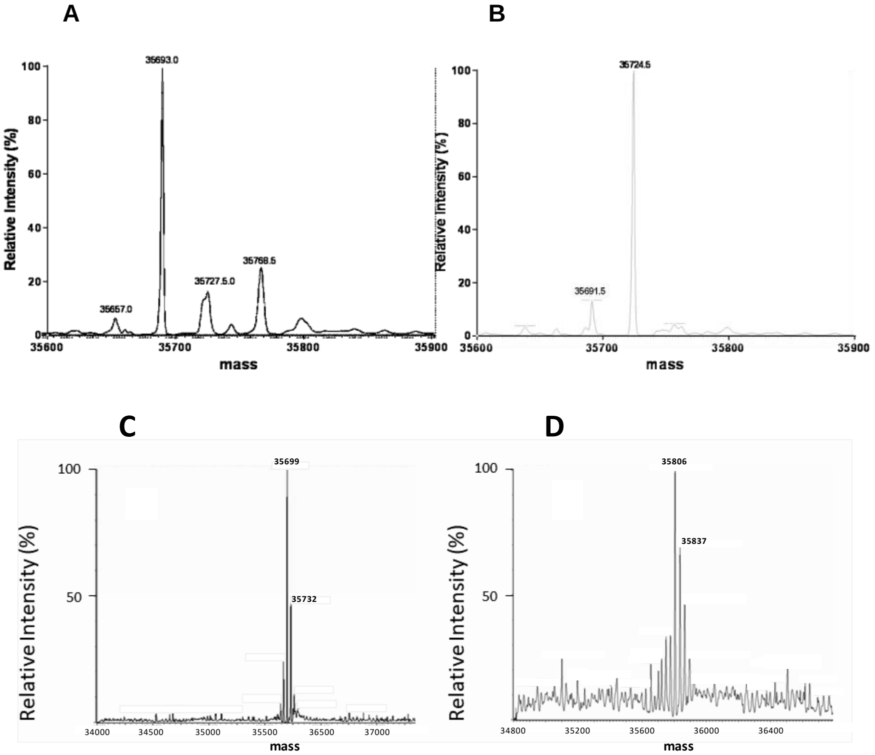

Native MW (Da)	H_2O_2-Oxidized MW (Da)	Δ (a.m.u)		
**35,693.0**	**35,724.5**	**31.50**	**Relative Intensity (%)**	
**Native-Incubated MW (Da)**	**H_2O_2-Oxidized-Incubated MW (Da)**		**Untreated (%)**	**H_2O_2-Oxidized**
**35,699.0**	**35,806.0**	**107.0**	**100.0**	**100.0**
**35,732.0**	**35,837.0**	**105.0**	**45.0**	**70.0**

Supplementary Figure S3. Related to 'Identification of redox-active cysteine intermediates'. Representative LC/ESI-QTOF mass spectrum of native and oxidized *r*-GAPDH. The predicted mass of *r*-GAPDH subunits is 35,648.7Da. (The mass average reconstruct m/z ratios of the major peaks are summarized in the table). **(A and B)** mass spectra of native and H_2O_2 oxidized *r*-GAPDH, in four separate experiments incubated for between 30 – 60min (incubation time-dependent on spectrometer availability). Immediately before dilution into the mobile phase, 5mM DTT was added to the samples prior to dilution into the mobile phase, and spectral data yielded Δ= +32.3±1.3Da (N=4). The mass increase after oxidation (31.5Da) is consistent with the addition of two oxygens/subunits (+32Da). **(C and D).** Mass spectrum of native and H_2O_2 oxidized plus IAA alkylation of *r*-GAPDH. Both samples were aerobically incubated for 2 weeks at 6°C. After the addition of DTT to the samples prior to dilution into the mobile phase, additional subunit peaks appeared. The comparison of m/z of the two major peaks in both native and H_2O_2-oxidized and alkylated was +33 and 31Da, respectively. The mass increase of both subunit species between native and H_2O_2-oxidized and alkylated *r*-GAPDH samples were +107 and +105Da for

the subunits with lower and higher masses, respectively. To account for these observations, following H_2O_2 oxidation, one of the four cysteines is initially modified to $C(SO_2H)$ (+32Da), while one is alkylated to carboxymethyl cysteine (+58.05Da). The disulfides are reduced by DTT after the aerobic incubation where $C(SO_2H)$ undergoes air oxidation to form $C(SO_3H)$ (+16Da) resulting in a total mass increase of 106.05Da. These results reinforce the biochemical and LC/MS/MS data that after eight mol H_2O_2 oxidizes eight mol cysteines/mol GAPDH, the oxidative modifications are homogeneously distributed in the homotetramer.

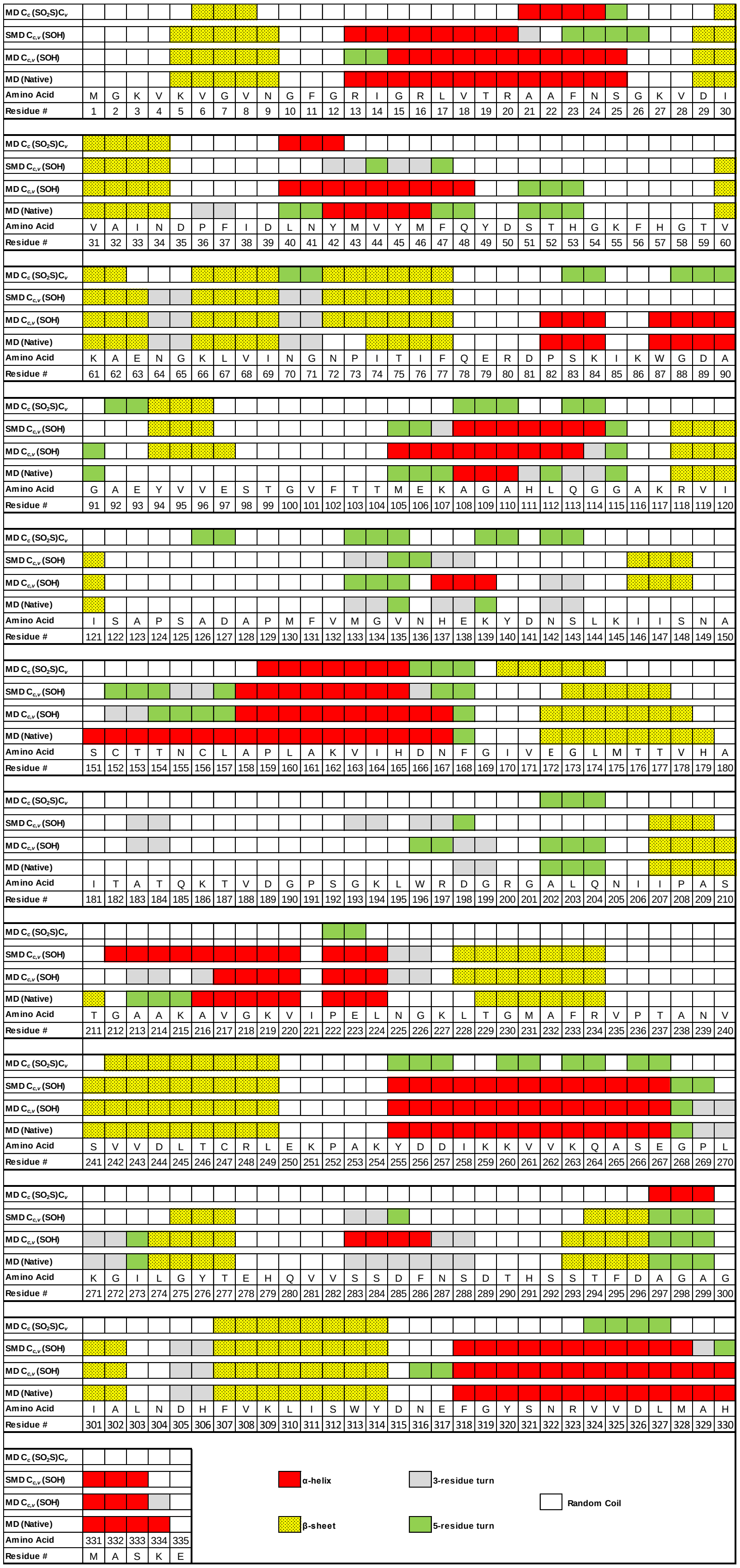
MD C_c $(SO_2S)C_v$
SMD $C_{c,v}$ (SOH)
MD $C_{c,v}$ (SOH)
MD (Native)
Amino Acid
Residue #
M G K V K V G V N G F G R I G R L V T R A A F N S G K V D I
1–30
V A I N D P F I D L N Y M V Y M F Q Y D S T H G K F H G T V
31–60
K A E N G K L V I N G N P I T I F Q E R D P S K I K W G D A
61–90
G A E Y V V E S T G V F T T M E K A G A H L Q G G A K R V I
91–120
I S A P S A D A P M F V M G V N H E K Y D N S L K I I S N A
121–150
S C T T N C L A P L A K V I H D N F G I V E G L M T T V H A
151–180
I T A T Q K T V D G P S G K L W R D G R G A L Q N I I P A S
181–210
T G A A K A V G K V I P E L N G K L T G M A F R V P T A N V
211–240
S V V D L T C R L E K P A K Y D D I K K V V K Q A S E G P L
241–270
K G I L G Y T E H Q V V S S D F N S D T H S S T F D A G A G
271–300
I A L N D H F V K L I S W Y D N E F G Y S N R V V D L M A H
301–330
331 332 333 334 335
M A S K E
α-helix
3-residue turn
Random Coil
β-sheet
5-residue turn

Supplementary Figure S4. Supports 'MD analysis of the secondary structure of oxidized GAPDH'. Linear representation of the full subunit sequence-structure resulting from the four MDS iterations. Secondary structural characteristics of *h*-GAPDH of the subunit domains after MDS and energy minimization analysis. The native structure (MD(Native) of 1u8f was modified by conversion of the catalytic (C152) and vicinal (C156) cysteine residues to their respective sulfenic acids following H_2O_2 oxidation (Steps 1 and 2). The resulting structure following perturbation of these oxidation steps [MDS ($C_{c,v}$S(OH)] was then subject to Steered Molecular Dynamics [SMD($C_{c,v}$S(OH)], resulting in the juxtaposition of the two sulfenic acid sulfur atoms within the van der Waal's contact distance for condensation. After the formation of the interchain thiosulfinic ester, the output from MDS demonstrated major structural changes within the subunit and dissociation of NAD^+ and solvent exposure of C244 and predicts subunit unfolding after the formation of the intrachain sulfur-sulfur bond.

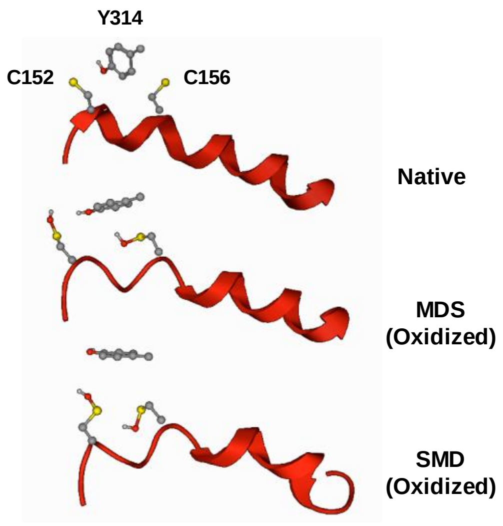

Native	Distance (Å)
C152/C156	8.58
C152/Y314 Centroid	4.98
C152/Y314-(OH)	4.9
C156/Y314 Centroid	4.45
C156/Y314-(OH)	3.2
C152--C156/Y314 π bond plane angle	90°
**Oxidized**	
C152/C156	7.22
C152/Y314 Centroid	5.83
C152/Y314-(OH)	3.4
C156/Y314 Centroid	4.2
C156/Y314-(OH)	4.1
C152--C156/Y314 π bond plane angle	84.4°
**Reaction Path**	
C152/C156	5.45
C152/Y314 Centroid	5.22
C152/Y314-(OH)	3.6
C156/Y314 Centroid	5.63
C156/Y314-(OH)	4.7
C152--C156/Y314 π bond plane angle	36.5°

Supplementary Figure S5. **Supports 'MD analysis of the secondary structure of oxidized GAPDH'.** Close-up view of the subunit active site α-helix rearrangement. The figure charts the displacement of the tyrosine Y314 hydroxyphenyl side-chain π-orbital centroid in the plane bisecting C_c(SH) and C_v(SH) (C152/C156) in the native enzyme. The angle between this plane, the hydroxyphenyl π-bonds, and the sulfur-sulfur center distances are shown after conversion of the native structure to C_c(SOH) and C_v(SOH) and subjecting the crystal structure to MDS and SMD (Reaction Path) with energy minimization. The resulting structure proves that (a) steric hindrance of Y314 is completely removed from the plane joining the two sulfur centers and (b) the sulfur centers are now within the van der Waals contact distance for sulfenic acid condensation.

Supplementary Note S1. A point of controversy is the disparity between the magnitude of the rate constant (*k*) for H_2O_2 oxidation of C_c(SH) repeatedly referenced in several articles, and that obtained when it is *actually measured*. At pH 7.0 at 37°C, we obtain a value of *k* of 9.4$M^{-1}s^{-1}$ for H_2O_2 oxidation of *p*-GAPDH. This compares favorably with the value published for H_2O_2 oxidation of *r*-GAPDH of 11.4$M^{-1}s^{-1}$ and 10$M^{-1}s^{-1}$ at pH 7.5 at 22°C[37,41]. References to literature values of 100 – 1,000 $M^{-1}s^{-1}$ has become regularly cited in reviews[10] and research articles[34]. We have examined several publication data sets where there is sufficient methodological information provided to estimate an approximate value of the bimolecular rate constant and invariably yields a value of ~10$M^{-1}s^{-1}$. This latter value is significant because of its similarity to the established value of H_2O_2 oxidation of aliphatic thiols such as cysteine[42] of ~10$M^{-1}s^{-1}$. GAPDH C_c(SH) is more reactive to alkylating agents compared to cysteine, and the conundrum of why the nucleophilicity of C_c(SH) toward different electrophiles is dissociated from its

basicity has been a subject of debate for many years and relates to complex ion-pairing within the active site[43]. Whatever the mechanism, the electronic environment of C_c(SH) in GAPDH *augments* its reactivity toward H_2O_2 in comparison to other electrophiles, a concept that is not generally accepted .

References

50. Strus, M.; Brzychczy-Wloch, M.; Gosiewski, T.; Kochan, P.; Heczko, P. B., The in vitro effect of hydrogen peroxide on vaginal microbial communities. *FEMS Immunol Med Microbiol* **2006,** 48, (1), 56-63.

37. Barinova, K. V.; Serebryakova, M. V.; Eldarov, M. A.; Kulikova, A. A.; Mitkevich, V. A.; Muronetz, V. I.; Schmalhausen, E. V., S-glutathionylation of human glyceraldehyde-3-phosphate dehydrogenase and possible role of Cys152-Cys156 disulfide bridge in the active site of the protein. *Biochimica et biophysica acta. General subjects* **2020,** 1864, (6), 129560.

41. Elkina, Y. L.; Kuravsky, M. L.; El'darov, M. A.; Stogov, S. V.; Muronetz, V. I.; Schmalhausen, E. V., Recombinant human sperm-specific glyceraldehyde-3-phosphate dehydrogenase: structural basis for enhanced stability. *Biochim Biophys Acta* **2010,** 1804, (12), 2207-12.

10 Hildebrandt, T.; Knuesting, J.; Berndt, C.; Morgan, B.; Scheibe, R., Cytosolic thiol switches regulating basic cellular functions: GAPDH as an information hub? *Biol Chem* **2015,** 396, (5), 523-37.

34 Peralta, D.; Bronowska, A. K.; Morgan, B.; Doka, E.; Van Laer, K.; Nagy, P.; Grater, F.; Dick, T. P., A proton relay enhances H2O2 sensitivity of GAPDH to facilitate metabolic adaptation. *Nat Chem Biol* **2015,** 11, (2), 156-63.

42. Barton, J. P.; Packer, J. E.; Sims, R. J., Kinetics of the reaction of hydrogen peroxide with cysteine and cysteamine. *J. Chem. Soc., Perkin Trans. II* **1973**, 1547–1549.

43. Harris, J. I.; Waters, M., Glyceraldehyde-3-phosphate dehydrogenase. In *The Enzymes*, Boyer, P. D., Ed. Academic Press: Boca Raton, 1976; Vol. Chapter 1, pp 1-49.
